# Targeting RNA with small molecules using state-of-the-art methods provides highly predictive affinities of riboswitch inhibitors

**DOI:** 10.1038/s42003-025-08809-y

**Published:** 2025-10-01

**Authors:** Narjes Ansari, Chengwen Liu, Florent Hédin, Jérôme Hénin, Jay W. Ponder, Pengyu Ren, Jean-Philip Piquemal, Louis Lagardère, Krystel El Hage

**Affiliations:** 1 Qubit Pharmaceuticals, 75014 Paris, France; 2 Department of Biomedical Engineering, The University of Texas at Austin, Austin, TX USA; 3 Université Paris Cité, CNRS, Laboratoire de Biochimie Théorique, Paris, France; 4 Department of Chemistry, Washington University in Saint Louis, Saint Louis, MO USA; 5 Sorbonne Université, Laboratoire de Chimie Théorique, UMR 7616 CNRS, Paris, France

**Keywords:** Drug discovery, RNA, Small molecules, Computational chemistry

## Abstract

Targeting RNA with small molecules represents a promising yet relatively unexplored avenue for the design of new drugs. Nevertheless, challenges arise from the lack of computational models and techniques able to accurately model RNA systems, and predict their binding affinities to small molecules. Here, we tackle these difficulties by developing a tailored state-of-the-art approach for absolute binding free energy calculations of RNA-binding small molecules. For this, we combine the advanced AMOEBA polarizable force field to the newly developed lambda-Adaptive Biasing Force scheme associated to refined restraints allowing for efficient sampling. To capture the free energy barrier associated to challenging RNA conformational changes, we combine machine learning-based collective variables with enhanced sampling simulations. Applying this computational protocol to a complex Riboswitch-like RNA target demonstrates quantitative predictions. These results pave the way for the routine application of free energy simulations in RNA-targeted drug discovery, thus providing a significant reduction in their failure rate.

## Abstract (web summary)

A novel state-of-the-art approach for absolute binding free energies of RNA-binding small molecules paves the way to the routine use of free-energy simulations in drug discovery targeting RNA systems, reducing failure rates.

## Introduction

RNA-targeting therapeutics offer an attractive alternative to reach traditionally undruggable proteins and expand the druggable target space in the human genome. Indeed, RNA can adopt three-dimensional (3D) structures, conferring varied functional roles in human biology as well as disease-related dysfunction. They are unique molecules capable of interacting with all three major forms of biological macromolecules: proteins, DNAs, and RNAs. Due to their diverse mechanisms of action, including gene silencing, splice modulation, and protein interaction, RNA therapeutics offer a versatile platform for drug development^1–4^.

Therapeutic strategies to target RNA include nucleotide-based agents such as (i) antisense oligonucleotides (ASOs), small interfering RNA (siRNA), and microRNAs (miRNAs) that can directly target messenger RNAs (mRNAs) and noncoding RNAs (ncRNAs) through Watson–Crick base pairing, (ii) clustered regularly inter-spaced short palindromic repeats (CRISPR) gene editing that can directly modify target RNA sequences to treat specific disorders, (iii) and small molecules that recognize RNA structures^2^. While ASOs and CRISPR editing have been invaluable to the field of chemical biology, their applications in therapeutics lacks efficient delivery strategies and presents significant adverse reactions^5,6^. These faced difficulties are due to their physico-chemical properties (size, charge,...), off-target effects, interactions with the immune system, rapid clearance and nuclease degradation. Alternatively, small molecules offer a promising alternative with potential for oral bio-availability and blood-brain barrier penetration. In particular, the extensive medicinal chemistry knowledge allows to systematically optimize pharmacokinetics and potency. Moreover, functional RNAs or RNA motifs are highly structured and form binding pockets or clefts that are accessible by small molecules.

Overall, targeting RNA with small molecules is emerging as a promising drug discovery approach. Such molecules interact with various target RNA substrates, including RNA structural motifs (such as hairpins, bulges, and internal loops), RNA enzymes (ribozymes), and specific RNA sequences^7,8^. Several binders have been identified and exhibit various modes of action (MOAs), from simple binding to direct cleavage or recruitment of endogenous nucleases. These molecules modulate diverse biological processes, such as inhibiting bacterial and viral translation (e.g., ribocil and a riboswitch in *Escherichia coli*^9^, 2-aminobenzimidazole derivatives and the hepatitis C internal ribosome entry site (IRES)^10^, respectively), and directing splicing by acting as molecular glues with cellular proteins (e.g., branaplam^11^ and the FDA-approved risdiplam^12^).

Challenges arise when tackling these systems throughout the drug discovery process and more specifically at the early stages, where the computer-aided drug design tools and simulation approaches are essential to characterize the target. Understanding its structural dynamics as well as its mechanism of action and interplay with other partners will guide the implementation of a pertinent inhibition or modulation mechanism. However, current methods face significant challenges in addressing the key obstacles associated with structure determination, accurate computational models, and reliable affinity prediction methods, which are the key elements required to guide the initial molecular design and rational drug optimization phases.

RNA’s chemical composition is distinct from proteins due to a highly electronegative surface potential associated to a limited buried surface area. It is highly charged, dynamic, and can adopt different conformations. In addition, divalent metal ions play an important role in its structural stability. Since RNA is also surrounded by ions and polarizable water molecules, it makes its targeting very challenging without having a reliable computational model that encompasses an accurate treatment of electrostatics^13,14^. This can be addressed by using second-generation force fields that account for many-body polarization effects^15,16^. Indeed, their inclusion in simulation play a key role in predicting biophysical properties governing the interactions between a target, its environment, and a potential drug candidate^17^. This is the case of AMOEBA, an advanced multipolar polarizable force field that employs atomic induced dipoles to include polarization effects and atomic multipoles, up to quadrupoles, to represent the anisotropy of permanent electrostatics^18,19^. These non-additive effects are essential for accurately ranking affinities of small molecules^13,20,21^. Although polarizable force fields were traditionally considered as computationally slow, limiting their early adoption, recent advances in computing hardware, especially GPUs (Graphics Processing Unit) have greatly improved the situation^22^. Further couplings with enhanced sampling techniques strongly increased the molecular dynamics (MD) timescales accessible to these simulations^14,23–28^.

Computing free energies of binding in silico has become a routinely used tool in drug discovery projects^29,30^. Among the existing techniques, alchemical ones, where the transformation of interest is made through a non-physical path, has proven to be both efficient and reliable, especially for relative free energies of binding (RBFE) between compounds^31^. Computing Absolute Binding Free Energies (ABFE) is more challenging but also more directly useful for drug discovery as it does not rely on the knowledge of a reference compound and is not limited to ligands sharing a similar binding mode. Computing binding affinities of small molecules bound to RNA or DNA with such methods remain a daunting challenge when compared to proteins^32–36^. Given the particular nature of RNA, it is considerably more sensitive to the protocol adopted than in the case of small molecule-protein complexes, especially when the ligand is large, charged, with highly flexible, elongated structure. In addition, both the RNA target and the ligand remain exposed to the solvent and the counterions, in contrast to ligand-protein complexes in which the ligand is in a partly buried protein cavity and interacts with only a limited number of “discrete” water molecules without intrusions from a counterion, which is common in RNA systems.

Furthermore, the shape of the ligand and its binding mode, i.e., intercalation or groove binding, will impact the overall protocol used to get ABFEs, especially in terms of the choice and the number of restraints used to keep the ligand in place during simulations and facilitate convergence^37,38^. Other aspects, such as the discretization of the alchemical path (i.e., the so-called “lambda windows” used in standard methods) and the sampling time are critical factors that should be taken into account when developing a tailored protocol within a well-defined workflow for ABFE calculations for RNA (and DNA) small molecule binders^27,32,39^. Moreover, the free energy barrier associated with the significant conformational change that may exist between the Apo and Holo structures of the target is notoriously challenging to estimate^40^.

A recently developed technique, the lambda-Adaptive Biasing Force (lambda-ABF) approach, grounded on λ-dynamics combined with a newly developed multiple-walker adaptive biasing force bias, bypasses the discretization of the alchemical path and enables efficient sampling of orthogonal degrees of freedom through the variable nature of the alchemical variable. In combination with a recently introduced restraint scheme^38^, distance-to-bound-configuration (DBC), it has shown improved performance compared to more standard methods^27^.

In this work, the inhibition of the hepatitis C internal ribosome entry site (HCV-IRES) IIa subdomain by nineteen 2-aminobenzimidazole derivatives is taken as a case study to address the aforementioned challenges and to illustrate how advanced and state-of-the-art computational techniques can be employed to compute the affinity of a class of small molecules for their RNA target with high accuracy.

**Fig. 1:**
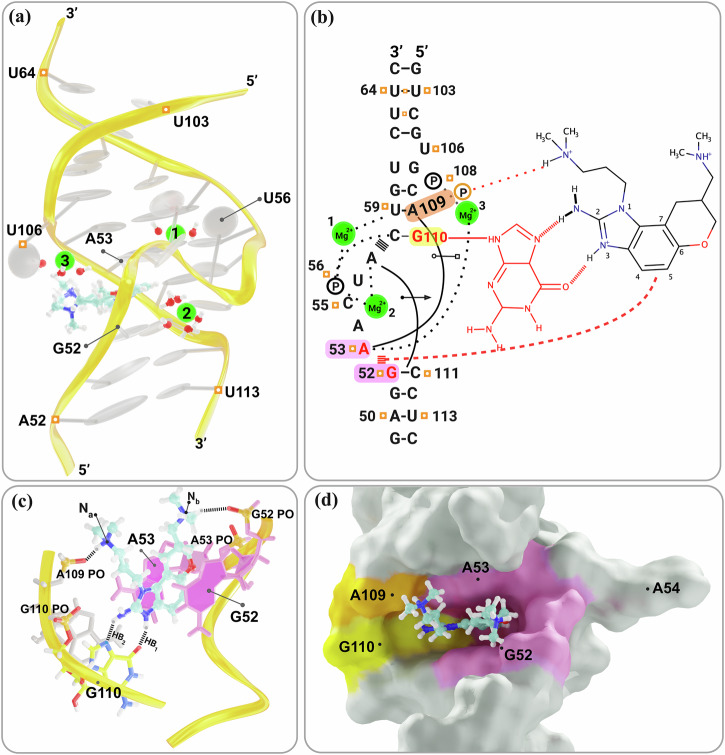
Structural features of the riboswitch-like RNA-Benzimidazole complex. **a** Overall view of the riboswitch-like RNA complex with Benzimidazole (ligand 4) after equilibration. The ligand is depicted in fluorescent blue. Three Mg^2+^ ions, shown in green, are coordinated by surrounding water molecules. **b** Schematic representation of interactions within the ligand-binding site. Hydrogen bonds are depicted by dashed lines. Non-Watson–Crick base pairs are shown with solid lines and symbols according to Leontis et al.^87^. Stacked lines ( ≡ ) denote base stacking and intercalation of the ligand, which is colored pink. Residues that interact with the benzimidazole are in red. **c** Detailed view of the ligand-binding site. The bases of G52 and A53, which form the intercalation site for the benzimidazole scaffold, are shown in pink. The purine base of G110 is colored yellow. Hydrogen bond interactions between G110 and the ligand are depicted with dashed lines and labeled as HB_1_ and HB_2_. Possible hydrogen bonds between the NH^+^ group of the ligand and A109 and G52 are also shown by dashed lines. **d** Surface representation highlighting the ligand-binding pocket. The interaction regions of A53 and G52 are colored pink, G110 is shown in yellow, and A109 is depicted in orange.

**Fig. 2:**
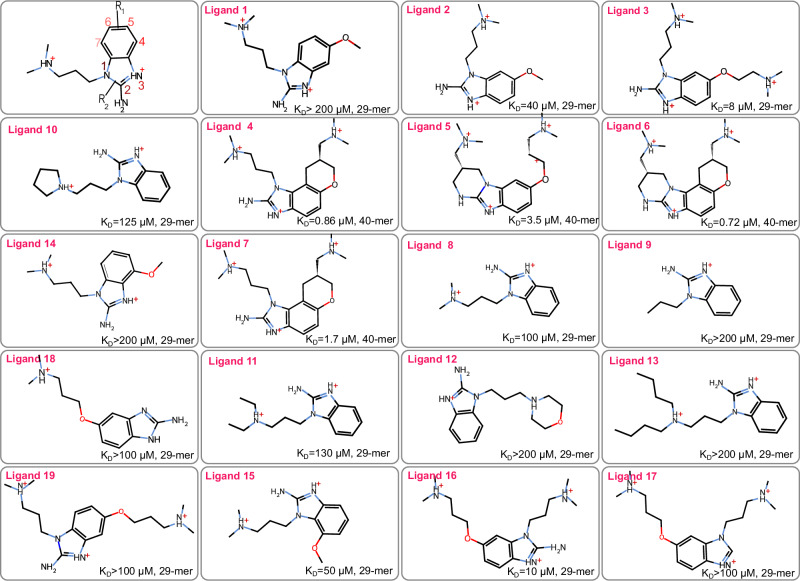
2D chemical structures of the nineteen studied translation inhibitors. 2D structure of the scaffold with common atoms labeled 1 to 7. The potential growing regions are highlighted as R_1_ and R_2_. For each ligand, the dissociation constant (K_*D*_) and the corresponding size of RNA are reported from Seth et al.^42^.

The HCV-IRES is essential for the initiation of viral protein synthesis and adopts well-defined folds that are potential targets for antiviral translation inhibitors. The 3D structure of the IRES domain IIa in complex with a benzimidazole translation inhibitor at 2.2 Å resolution (PDB ID 3TZR) was determined and provides insights into inhibiting its function^10,41^. This system is chosen for its complexity that makes it one of the most challenging systems for atomistic simulations and absolute binding affinity calculations. First, the ligand-binding site has an intricate architecture, organized by a metal spine at the back of the cavity. Additionally, it contains three magnesium ions as intrinsic structural components (Fig. 1). Moreover, the inhibitors are 2-aminobenzimidazole derivatives containing 2 to 3 positive charges, as well as several aromatic and non-aromatic cycles with lengthy arms (see Fig. 2)^42^. Finally, the comparison of the structures of bound and unbound RNA shows that the RNA undergoes a dramatic ligand-induced conformational adaptation from an L-shaped to an extended form, creating a deep pocket that resembles the substrate binding site in riboswitches. Also, the aforementioned three magnesium ions undergo adaptive reorganization upon binding of the benzimidazole ligand^41^.

In order to comprehend the binding mode of these inhibitors and reproduce their binding affinities, we have resorted to a computational model that accounts for the underlying effects of polarization and electron anisotropy, AMOEBA^18,43,44^, recently parameterized for DNA and RNA using quantum chemistry (QC) methods^19^ and tested in several oligonucleotide simulations^32,33,45^. We have also used the massively parallel GPU-accelerated molecular dynamics Tinker-HP software package^22,23,46,47^ coupled to the Colvars library^27,48^. We have put in place a novel state-of-the-art approach for ABFE calculations tailored for riboswitch-like RNA using the lambda-ABF scheme^27^ combined with positional, orientational, and conformational restraints^38^. We also propose a strategy to estimate the free energy difference associated to the conformational change between the Apo and the Holo structure of the riboswitch through advanced simulation techniques leveraging machine-learned collective variables (CVs)^49^.

## Results

In this section, we first assess the binding mode of the studied small molecules to their RNA target, followed by computing their binding affinities. Finally, we explore the conformational changes between the Apo and Holo states of the RNA.

### Binding mode analysis

The RNA architecture of the ligand-binding pocket consists of the phosphate of U56, which is rotated into the RNA helix major groove, and two magnesium ions anchored between the phosphate oxygen atoms and the bases of A57 and U59 (Fig. 1b). The extreme contortion of the RNA backbone that directs the U56 phosphate into the helix interior is further pinned in place by hydrogen bonds between the C55 phosphate and 2’-hydroxyl groups of the flanking residues A54 and U56. The elaborate network of backbone and metal interactions forces the base of A54 to project away from the RNA whereas residues C55 and U56 tightly pack in the RNA helix major groove as well as against the backbone of C58 and U59 (Fig. 1d). The third magnesium ion closes part of the solvent exposed part of the ligand site by bridging the RNA strands with interactions at the base of A53 and the C108 phosphate. The top and bottom part of the binding pocket are stabilized by base triples which form through cross-bracing interactions along the RNA helix with A53 docking at the Hoogsteen edge of A109 and A57 interacting with the sugar edge of C111. Additional hydrogen bonds stabilize the distorted internal loop in the benzimidazole complex and include interactions of the U56 phosphate with the C58 base and of the C55 2’-hydroxyl with the A53 base (Fig. 1b).

**Fig. 3:**
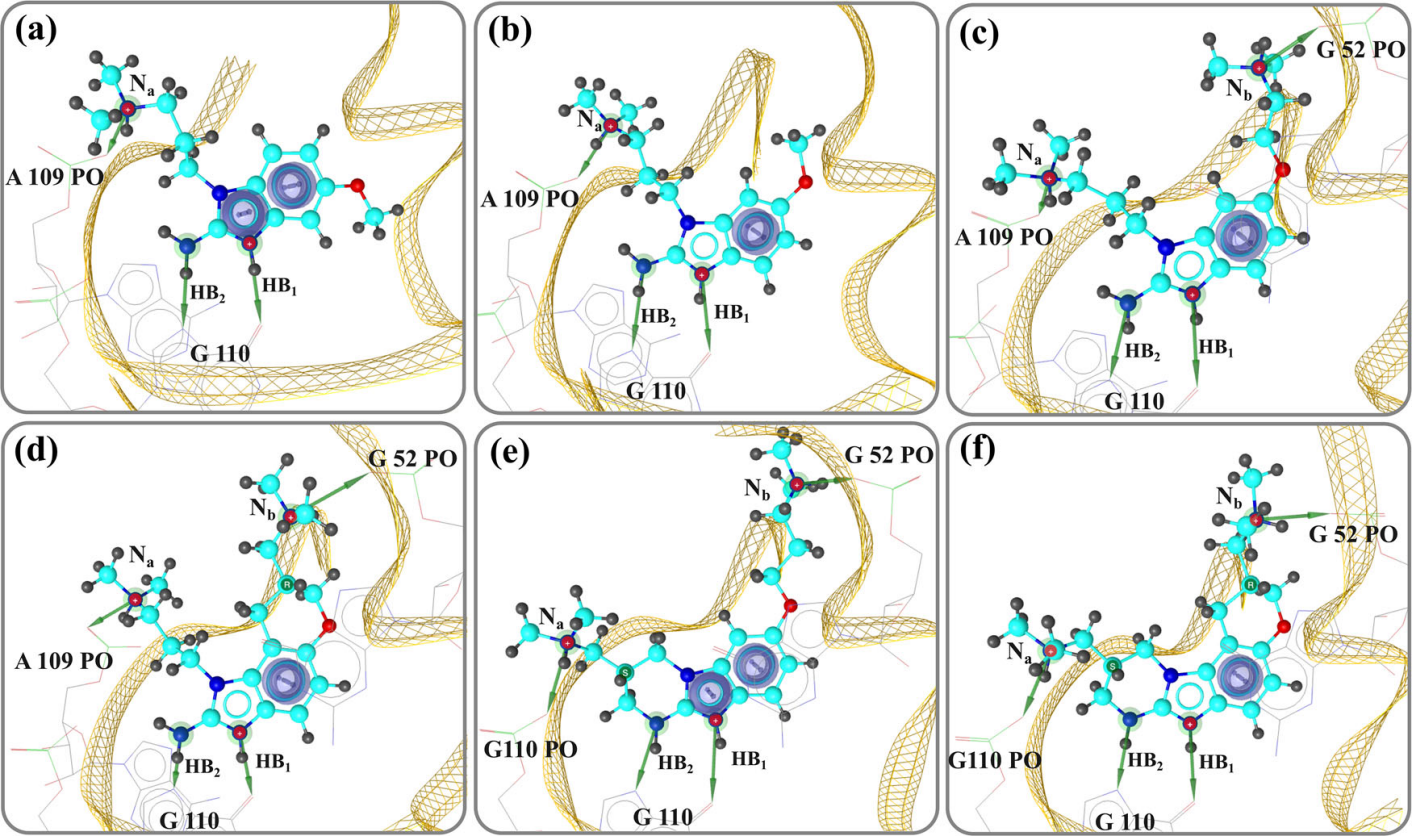
3D representation of the binding modes of six representative ligands and their interactions with the environment. **a** – **f** show the binding modes of Ligands 1 to 6, respectively. Ligands are depicted in licorice style and colored in cyan, while the phosphate backbone is represented by a yellow meshed ribbon. Green arrows indicate hydrogen bond donors, and purple circles indicate *π*-*π* stacking interactions between the G52, A53 bases and the ligand. The positively charged nitrogen (N) atoms are shown in red with a superscript (+). The chirality labels are displayed as **S** or **R** for chiral centers.

Understanding this intricate architecture is key to analyzing how the ligands interact with the RNA structure. It is well-known that the contribution of hydrogen bonding and stacking interactions are predominate in RNA recognition^50^. Following the detailed relaxation process described in the simulation setup, we analyzed the binding modes obtained from the 40 ns MD simulations. Figure 3 illustrates the binding modes of six representative ligands, out of nineteen, within the target RNA. These ligands, ligand 1 to 6, cover different ranges of affinities and include a non-binder, ligand 1 (see Fig. 2). Three primary interactions are identified as crucial for stabilizing the ligand within the binding pocket: (i) a *π*-*π* stacking interaction occurs between the ligand and the nucleic bases G52 and A53, (ii) two hydrogen bonds (labeled HB_1_ and HB_2_) formed between the central core of the ligand (benzimidazole) and G110 (see Fig. 1c), and (iii) an additional ionic interaction is established between the NH^+^ moiety of the dimethylazanium group at the end of each arm of the ligand and the non-bridging phosphate oxygen atoms (PO) of A109 (or G110) and the PO of G52 (or A53) on the opposite strand (depicted as N_*a*_ and N_*b*_, respectively in Fig. 3).

**Fig. 4:**
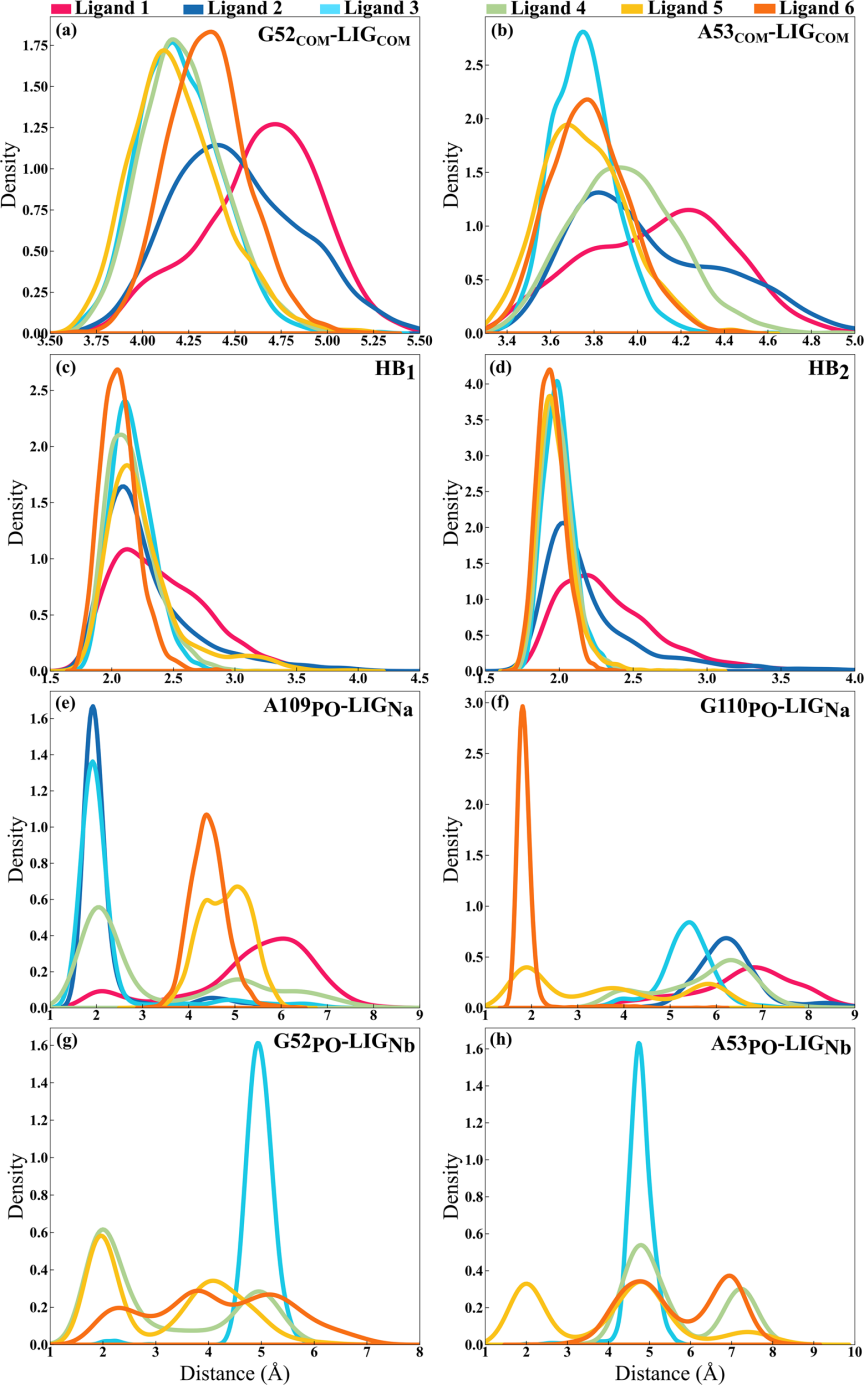
Normalized distributions of key interaction quantities for six representative ligands. The distributions for distances between **a** G52_COM_ and LIG_COM_, **b** A53_COM_ and LIG_COM_, **c** HB_1_ distances, **d** HB_2_ distances, distances between **e** A109_PO_ and LIG_Na_, **f** G110_PO_ and LIG_Na_, **g** LIG_Nb_ and G52_PO_, **h** LIG_Nb_ and A53_PO_. The colors represent different ligands as indicated in the legend.

With these key interactions established, their evolution as a function of time was computed throughout the trajectory and for each ligand; the normalized distributions are shown in Fig. 4. For the *π*-*π* stacking interactions, we computed the distance between the center of mass (labeled as COM) of the purine rings of G52 and A53, and the COM of the benzimidazole scaffold of the ligand (refered to as LIG_COM_ in Fig. 4). For the distances between G52_COM_ and LIG_COM_, the results reveal that for most ligands, the distribution is narrow and centers around 4.1 to 4.4 Å, consistent with the typical *π*-*π* interaction range of 3.3 to 4.4 Å. In contrast, for ligands 1 and 2, the distribution is broader, with ligand 1 centering around 4.8 Å while showing a shoulder at 4.0 Å. As for ligand 2, the distribution centers around 4.3 Å with a shoulder at 5 Å. This is concomitant with the fact that ligand 1 is a non-binder, while ligand 2 is the weakest binder in the list. Similarly, the distribution of distances between A53_COM_ and LIG_COM_ shows a comparable trend for all ligands. Here, the distribution also centers around a narrow range, specifically between 3.6 and 3.8 Å for ligands 3 to 6. This indicates that the overall trend in interaction distances is consistent with the *π*-*π* stacking interactions observed with G52_COM_, albeit with a slightly shorter average distance for the interactions involving A53_COM_.

Next, the hydrogen bond interactions, HB_1_ and HB_2_, were analyzed. For these interactions, ligand 1 exhibits a broader distribution, suggesting a weaker and more transient interaction. In comparison, ligand 6 exhibits a narrower distribution, centered around 2 Å with the highest intensities, reflecting strong and consistent interactions. Ligands 3, 4, and 5 show a peak similar to ligand 6 for HB_2_, although with slightly lower intensity for HB_1_. Ligand 2 shows a similar peak as the other ligands; however, it has a tail at low probability, suggesting occasional weaker interactions.

Finally, regarding the ionic interaction, the N_*a*_ atom of ligands 2, 3, and 4 forms a hydrogen bond with the phosphate (PO) atoms of A109, as indicated by a sharp peak around 2 Å. Ligand 6 is unique in forming an HB with the PO atom of G110. For the second arm of the ligand (N_*b*_ atom), ligands 4, 5, and 6 alternate between the PO atom of G52 and that of A53, as evidenced by multiple peaks in the distribution, one of which is centered around 2 Å. Ligands 1, 2, and 3 are also the only ligands that do not form a HB with the PO atom of G52, nor with A53, except for a rare HB with the PO atom of G52 at 2 Å for ligand 3.

### Binding affinity calculations

To accurately predict the binding affinities of extended charged ligands (bearing 2 to 3 charges) bound to a long 29-mer riboswitch-like RNA with three structural Mg^2+^ ions as intrinsic components in the vicinity of the binding pocket, we have developed a specific protocol utilizing the lambda-ABF method and DBC restraints.

As free energy is a state function, the computed Δ*G*s are fundamentally independent of the intermediate states sampled during an alchemical simulation. This allows for tailored modifications in these states to ensure better sampling and convergence as it is routinely done in restraining the ligand to keep it in the binding site while scaling down its non-bonded interactions with the environment. As mentioned earlier, different types of restraints exist that can either be positional or positional and orientational. However, these two methods suffer from distinct limitations^27,38^, especially when considering elongated ligands containing two flexible arms. What is required in such systems is positional, orientational as well as conformational restraints, that can be defined through the newly developed DBC collective variable, which is the root mean square deviation (RMSD) of the ligand in the moving reference frame of the binding site^38^. Therefore, to facilitate sampling and maintain the ligand in the binding site, we employed DBC restraints between the benzimidazole ring of all ligands and heavy atoms from the RNA binding site. The selected atoms are highlighted in Supplementary Fig. S1.

Besides, it is well known that starting alchemical simulations from Holo structures may lead to incomplete relaxation towards the Apo structure^51^. This incomplete relaxation can result in a significant free energy difference, potentially causing errors of several kcal/mol in the final prediction. Typically, to address this, all predicted affinities are shifted by a common value to account for this discrepancy^51^. This adjustment assumes that all systems experience the same degree of relaxation from the Holo to the Apo state. To ensure the validity of this assumption, facilitate the sampling and maintain structural stability of the RNA-ligand complex, we implemented slight positional restraints with a force constant of 1 kcal/mol/A^2^) on the phosphate (P) atoms of RNA and Mg^2+^ ions. These adjustments were designed to avoid impacting the fully bound state, ensuring the integrity of the complex throughout the simulations. This approach allows us to independently estimate and account for the relaxation effect, thus improving the accuracy of our absolute binding affinity calculations.

**Fig. 5:**
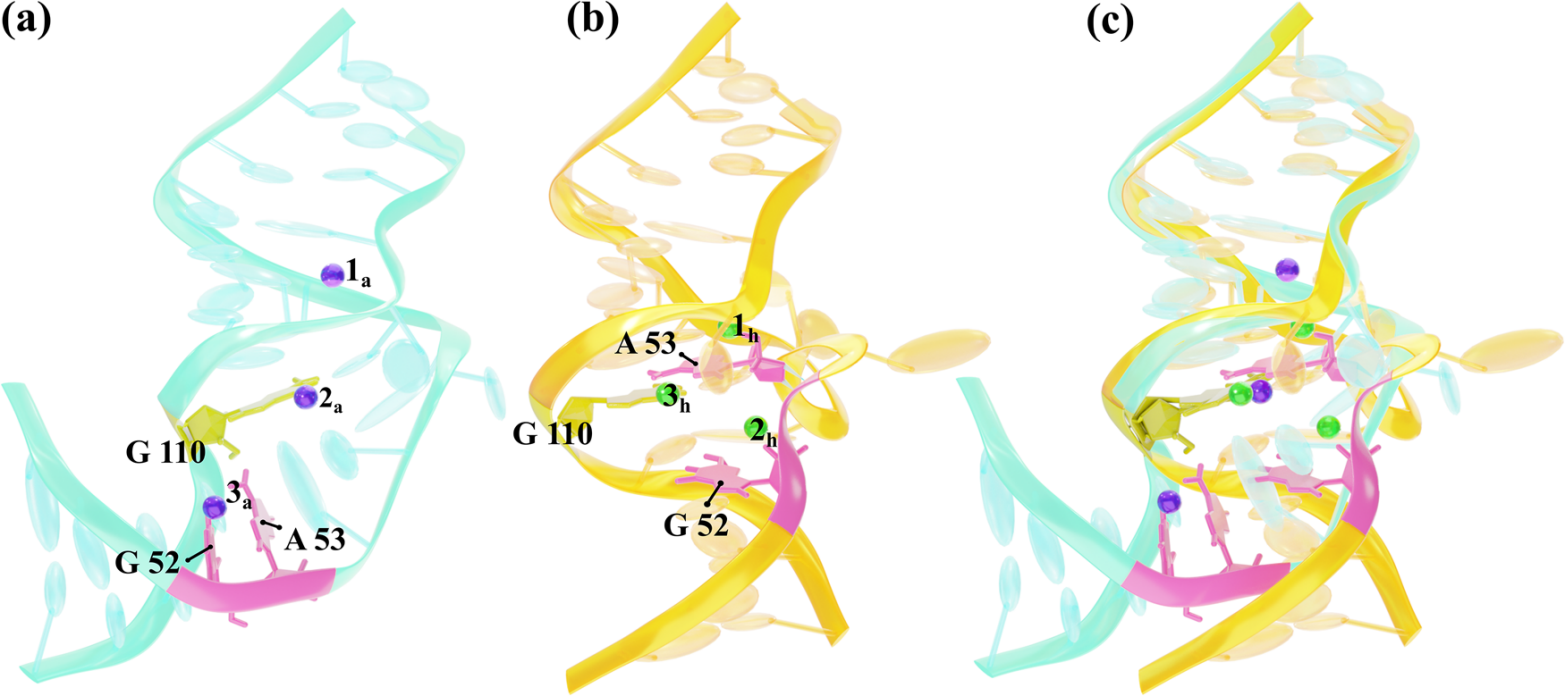
Conformational differences between Apo and Holo forms of RNA. **a** L-shaped structure of the Apo state (PDB ID: 2nok) and **b** straight structure of the Holo state (PDB ID: 3tzr). **c** Alignment of the Apo and Holo structures. The positions of Mg^2+^ ions in each structure are illustrated, denoted as *i*_*a*_ and *i*_*h*_ for the Apo and Holo states respectively, where *i* = 1 − 3. The key bases within the binding pocket are highlighted, with G110 in dark yellow and G52 and A53 in pink.

In the case of our system, it is known that benzimidazole inhibitors can induce a conformational change characterized by a widened interhelical angle in the IRES subdomain IIa^10,41,52^. This conformation facilitates the undocking of subdomain IIb from the ribosome, leading to the inhibition of IRES-driven translation in HCV-infected cells. As illustrated in Fig. 5, the L-shaped RNA structure in the Apo state is known to adopt a straighter configuration^10,41,52^. Apart from the restructuring of several nucleic bases, this conformational change also includes a rearrangement of Mg^2+^ ion positions (Fig. 5a, b). To mitigate any potential bias from such known conformational changes, we applied the restraints described earlier. This approach helps ensure that such known rearrangements are properly accounted for, thereby improving the reliability of our ABFE calculations.

To obtain the alchemical free energy surface for each system at *T* = 300 K, we ran three replicas, each utilizing four walkers. Calculations were conducted in both the complex (bound state) and solvent (ligand in the bulk) phases. For the complex phase, simulations ran for ~50 ns for both van der Waals (vdW) and electrostatic (ELE) legs. In contrast, a 20 ns run was enough for convergence in the solvent phase for both VDW and ELE components. Raw data from each replica of the six representative ligands with varying ranges of affinities (Ligand 1 to 6) are provided in the Supplementary Information, Section II and Supplementary Tables S2 and S3. Convergence plots are also provided as Supplementary Figs. S2 to S25. The free energy cost associated with the release of the DBC restraint is readily computed in the gas phase using TI^38,53^. This is achieved by gradually releasing the restraint to a compatible harmonic distance restraint, the cost of which can be computed analytically (see Supplementary Table S1).

As described in the Methods section, we have employed two distinct setups for ABFE calculations, one consisting of a neutralized system (Neut.) for all ligands and a second including a physiological ion concentration (Phys.) for the six representative ligands (Ligand 1 to 6). The computed ΔG values of all ligands in neutralized conditions are reported in Table 1 and compared with the experimental data. Supplementary Table S4, compares the computed ΔG values in neutralized condition (Δ**G**_Comp−Neut_) and in the presence of physiological ion concentration (Δ**G**_Comp−Phys_) for the six representative ligands. The raw absolute binding free energies (Δ**G**_**Comp**−**raw**_) consistently exhibit stronger values (favorable) compared to the experimental results. In order to account for the Apo-Holo conformational change, the raw binding free energy (Δ**G**_**Comp**−**raw**_) is converted to the final binding free energy Δ**G**_Comp_ by adding a constant shift, which is calculated as follows ${{{\boldsymbol{\Delta }}}}{{{{\bf{G}}}}}_{{{{\rm{shift}}}}}=\frac{{\sum }_{{{{\bf{i}}}}}{{{{\bf{G}}}}}_{{{{\bf{i}}}}}^{{{{\rm{Exp}}}}}-{\sum }_{{{{\bf{i}}}}}{{{{\bf{G}}}}}_{{{{\bf{i}}}}}^{{{{\rm{Comp}}}}}}{N}$^51^, so that the mean of computed free energies is equal to the mean of experimental free energies. This shift yield a value of 10.31 ± 1.1 kcal/mol for the neutral setup taking into account all ligands. Taking into account the six representative ligands, a value of 10.35 ± 0.91 kcal/mol is obtained for the neutral setup and 11.33 ± 1.19 kcal/mol in the presence of physiological ion concentration. Despite this slight difference, the results are consistent within the error margins.

**Table 1:** Experimental and computed Δ**G** values for each ligand

Ligand	K_D_(*μ*M)	$\Delta {{{{\rm{G}}}}}_{{{{\rm{Exp}}}}}$ (kcal/mol)	ΔG_Comp_(ΔG_Comp−raw_) (kcal/mol)
1	> 200	> − 5.04	− 0.12 ( − 10.43) ± 0.92
2	40	− 6.01	− 6.65 ( − 16.96) ± 0.68
3	8	− 6.96	− 7.30 ( − 16.88) ± 1.54
4	0.86	− 8.27	− 7.17 ( − 17.48) ± 0.65
5	3.50	− 7.44	− 7.25 ( − 17.56) ± 0.81
6	0.72	− 8.39	− 9.64 ( − 19.95) ± 1.54
7	1.70	− 7.88	− 7.26 ( − 17.57) ± 1.78
8	100	− 5.45	− 4.49 ( − 14.79) ± 0.56
9	> 200	> − 5.04	− 3.56 ( − 13.87) ± 0.53
10	125	− 5.32	− 7.30 ( − 17.61) ± 0.53
11	> 200	>-5.04	—
12	> 200	> − 5.04	− 0.71 ( − 11.01) ± 1.28
13	> 200	> − 5.04	1.07 ( − 9.24) ± 1.38
14	> 200	> − 5.04	2.92 ( − 7.39) ± 1.66
15	50	− 5.86	− 6.73 ( − 17.04) ± 1.03
16	10	− 6.82	− 5.32 ( − 15.63) ± 1.50
17	> 100	> − 5.04	− 2.81 ( − 13.12) ± 1.11
18	> 100	> − 5.04	1.40 ( − 8.90) ± 0.92
19	> 100	> − 5.04	3.04 ( − 7.27) ± 0.83

Computed Δ**G**_**Comp**_ values are reported under neutralized conditions. The computed Δ**G**_**Comp**_ and errors represent the mean and standard error of the mean from three replicas for each ligand (*n* = 3). Raw computed Δ*G* values, Δ**G**_**Comp**−**raw**_, are shown in parentheses. For Ligands 4, 5, 6 and 7 the experimental ${{{\boldsymbol{\Delta }}}}{{{{\bf{G}}}}}_{{{{\bf{Exp}}}}}$ is measured on a 40-mer RNA, while for the rest, the 29-mer is used. All computed ΔG were measured using a 29-mer construct. ΔG values are reported in kcal/mol. **K**_**D**_ values are reported in *μ* M.

**Fig. 6:**
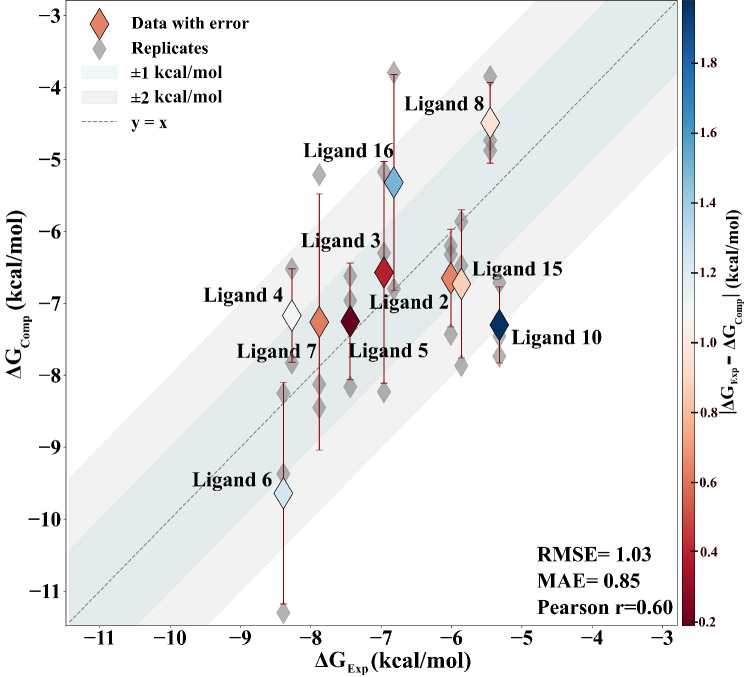
Experimental vs computed ΔG values. Computed ΔG and errors are the mean and standard error of the mean from three replicas for each ligand in the neutralized complex (*n* = 3). The dark shaded region spans  ±1 kcal/mol; the lighter region spans  ±2 kcal/mol. The color bar represents the absolute value difference between experimental and computed values. Pearson r, RMSE, and MAE are reported in the right bottom corner of each plot. The non-binders are not shown in the plots.

The correlation plots are illustrated in Fig. 6 and Supplementary Fig. S26. Upon including the systematic shift between the calculated and experimental binding free energies to accommodate the Apo-Holo protein reorganization free energy, we observe a correlation between computed and experimental results across the entire dataset of 0.6 (Pearson *r*), with a root mean square error (RMSE) of 1.03 kcal/mol and a mean absolute error (MAE) of 0.85 kcal/mol for the neutralized setup (Fig. 6). It’s worth noting that ligands 1, 9, 11, 12, 13, 14, 17, 18, 19 were excluded from the analysis as they are predicted to be non-binders in agreement with the experimental measurement which showed no binding at 100 or 200 μ M. As for ligand 11, also a non-binder, it leaves the pocket after 70 ns of plain MD, and hence was not considered for ABFE calculations. These results show a solid reproduction of the binding affinities and a good ranking, with ligand 6 and ligand 4 identified as the most potent and ligands 1, 9, 11, 12, 13, 14, 17, 18, 19 as non-binders. Taking into account only the six representative ligands, we observe a similar correlation between the two set-ups: a correlation of 0.7, with an RMSE of 0.81 kcal/mol and an MAE of 0.70 kcal/mol for the neutralized setup (Supplementary Fig. S26a); a correlation of 0.7 with an RMSE of 1.05 kcal/mol and an MAE of 0.84 kcal/mol for the set-up with a physiological concentration of salt (Supplementary Fig. S26b). Overall, both setups show similar satisfactory results.

Even though the experimental K_*D*_ measurements were done with 2 different constructs, this shouldn’t have a large effect on the affinities, as seen for the example of ligand 3, where K_*D*_=10 μ M in 40-mer compared to 17 μ M in the 29-mer^42^, which translate into 0.3 kcal/mol of difference, or on ranking the selected compounds in our study, it nonetheless might slightly effect the observed correlations. This is also without considering the error on the experimentally measured affinities.

#### Performance

To illustrate the computational cost required for one ABFE calculation, we have conducted benchmarking tests of lambda-ABF simulations on three different GPU architectures: NVIDIA H100, A100 and V100. This was done on the complex phase which is the most computationally expensive one. The performance on different GPU types is reported in Supplementary Table S5. As expected, it is lower on A100 and V100 GPUs due to differences in computational power and memory bandwidth. The performance on H100 GPUs is 1.5 times faster than A100 and 2.4 times faster than V100 GPUs. Details are provided in Section IV of Supplementary Information. All in all, the total amount of simulation time for the ABFEs ran in the present study encompasses more than 28 μs.

### Apo-Holo conformational change

As mentioned in the above subsection, the ΔG_shift_ resulting from the energy difference between the computed ΔG and experimental values is estimated to be between 10.31 and 11.35 ± 1.0 kcal/mol, primarily reflects the Apo-Holo conformational change. To substantiate these findings, employing more direct computational methods is crucial. Given the substantial free energy barrier associated with the Apo-Holo transition, capturing this difference accurately requires advanced sampling techniques. As illustrated in Fig. 5, the Apo-Holo conformational change involves complex collective motions that are challenging to capture with standard methods. Simple CVs, such as distances or dihedrals, are insufficient for this system. Instead, more advanced machine learning-based CVs can be employed. To address this, we employed the OPES-Explore^54,55^ enhanced sampling method in conjunction with Deep-Linear Discriminant Analysis (Deep-LDA)^49^, a machine learning-based technique specifically designed to identify effective CVs for complex transitions.

To identify the Deep-LDA CV, we conducted an unbiased simulation of 40 ns from both Apo and Holo structures, incorporating 47 descriptors that included distances and angles between various P atoms and three Mg^2+^ ions. The setup and training of the neural network is detailed in Supplementary Information Section V. Subsequently, we performed exploratory OPES simulations, each lasting 10 to 15 ns, with applied biases ranging from 2 to 15 kcal/mol on the Deep-LDA CV. Notably, a transition from the Apo to the Holo state was observed around 10 ns when the applied bias exceeded 13 kcal/mol (see Supplementary Figs. S27 and S28), which aligns well with the energy change estimated from our lambda-ABF calculations. However, the conformational changes of specific bases is not captured by the P and Mg atoms in our Deep-LDA CV as well as the still approximate nature of the OPES strategy.

Despite the inherent challenges in achieving precise free energy calculations given the system’s complexity, our approach provides a valuable qualitative approximation of the free energy barrier associated with the Apo-Holo conformational change.

## Discussion

The complexity and dynamic nature of RNA, combined with its critical role in various biological processes, makes it a very difficult target in drug discovery. However, with these challenges, it becomes essential to harness the latest computational techniques in order to predict the binding affinities between drug candidates and the target of interest with high precision.

In this study, we present a state-of-the-art approach for calculating ABFEs of RNA-binding small molecules for application in drug discovery, showcasing its effectiveness in a challenging RNA system: the HCV-IRES riboswitch-like RNA. This system presents a highly charged and flexible RNA structure, an intricated binding site with three magnesium ions as intrinsic components, ligands with multiple positive charges and several aromatic and non-aromatic cycles with lengthy arms, as well as a substantial ligand-induced conformational change between the Apo and Holo states.

All in all, by combining the computational performance of the GPU-accelerated Tinker-HP package to enhanced sampling and machine learning, while leveraging the accuracy of the AMOEBA force field, we are able to model challenging drug discovery targets such as RNA and to compute absolute binding affinities to guide drug design with the lambda-ABF methodology. This synergy not only overcomes the limitations of available free energy methods but also establishes a new standard in computational modeling of RNA-ligand interactions. The present work represents a new achievement in computational methodologies for drug design, where the complexities of RNA and other challenging targets can be addressed with unparalleled accuracy and speed, thus guiding the development of RNA-targeted therapeutics with precision and confidence.

## Methods

### System preparation

The initial input for the simulation comprises the 3D X-ray structure of riboswitch-like RNA in the presence of a benzimidazole derivative (ligand 4 shown in Figs. 1 and 2d), obtained from the Protein Data Bank (PDB ID: 3TZR, resolution 2.21 Å). Using this structure as a starting point, we generated the input for all other ligands illustrated in Fig. 2. Concerning, ligands 5 and 6 which are diastereomers they were designed to be conformationally restricted benzimidazoles to increase the binding, and the desired conformation was separated by HPLC^42^. Hence, for our simulations the desired 3D conformation was generated based on ligand 4 (SS0 in 3TZR). For consistency, we used a 29-mer RNA, as depicted in Fig. 1b, with all compounds. In the work of Seth et al.^42^, ligands 4, 5, 6 and 7 were measured with a 40-mer construct, while ligands 1, 2, and 3 were measured with the 29-mer construct. However, this does not significantly affect the K_*D*_ value or the ranking, given that the results for ligand 3 reported in Tables 2 and 3 of Seth et al.^42^ are consistent. Ligand 3 was tested with both the 29-mer and 40-mer RNA and it was found to have a slightly stronger binding to the 29-mer (K_*D*_ = 10μ M in the 40-mer compared to 17 μ M in the 29-mer). Given the experimental measurement error, we expect the results to be within the same range.

### AMOEBA force field parameters

The systems studied here are highly charged and dynamic, while being surrounded by ions and polarizable water molecules. To accurately model these systems, we employed the AMOEBA force field, which accounts for polarization and anisotropy through a multipolar representation of electrostatics. AMOEBA has been recently parameterized for DNA and RNA based on QC methods and validated in several oligonucleotide simulations^19^. Additionally, we used the Tinker-HP software on GPUs^46^ for efficient computational performance.

#### Generating AMOEBA force field parameters for small molecules

Ligand parameterization is performed using QC calculations and the Poltype package^56^ which facilitate the automated generation of AMOEBA parameters. The process begins with an SDF file defining the atom coordinate, bond order, ionic state, etc. Prior to geometry optimization, one conformation was selected from 500 conformers generated by the RDKit tool. Structure characteristics including intra-molecular hydrogen bonds, radius of gyration, solvent-accessible surface area were considered during the selection. Key torsion angles were kept frozen to the values of the selected conformer during subsequent MP2/6-31G(d) geometry optimization to maintain the preferred conformation. Two single-point (SP) calculations were performed on the optimized structure using the Psi4 software^57^: MP2/6-311G(d,p) for low-level and MP2/aug-cc-pvtz for high-level. Electron density from the low-level SP was used with the GDMA program^58^ (with switch value 0) to generate initial atomic multipoles (charges, dipoles, and quadrupoles). Electron density from high-level SP was employed to calculate the electrostatic potential (ESP) on defined grids surrounding the molecule. The POTENTIAL program within Tinker 8 software^59^ further refined the dipoles and quadrupoles by fitting them to reproduce the ESP. Valence (bond, angle, stretch-bend, and opbend), vdW, and torsion terms were matched using SMARTS patterns and assigned from published parameter databases^60^. Notably, vdW parameters for aromatic rings were assigned using those from a previous study^61^ due to their improved interactions with DNA and RNA. For any missing torsion parameters, a conventional spin-and-fit procedure was used. This involved restrained optimization using the xtb program^62^ with GFN-2 model^63^ and SP energy calculations at *ω*b97x-d^64^/6-311+G(d) level in Psi4 software^57^. To minimize computational cost and potential steric clashes during rotating the bond of the whole ligand, a fragment molecule containing the fitted torsions was extracted from the ligand and used for torsion parameter fitting.

### MD simulations

All systems were neutralized by adding the appropriate number of counterions (K^+^) in a water box of 80 Å × 80 Å × 80 Å such that the distance between the FLAP and its periodic images is at least 16 Å. The systems contain  ~ 52,000 atoms and were subject to NVT and NPT simulations for equilibration before production.

The systems underwent initial minimization using Tinker-HP “minimize” program, followed by a gradual heating process in 250 ps intervals. The temperature was increased from 50 K to 100 K, 200 K, and 300 K under the NVT ensemble, with RNA-ligand atoms restrained using a force constant of 10 kcal/mol/A^2^). During this step, MD simulations were conducted using the Verlet integrator with a time step of 1.0 fs. Subsequently, the systems were equilibrated in the NPT ensemble at 1 atm using the same restraints with force constants of 10 and 5 kcal/mol/A^2^), each for 0.5 ns, using the RESPA integrator^65^ and a time step of 2 fs. To relax the water molecules around the ions at 300 K, a 10 ns restrained MD simulation was run for each system with a force constant of 1 kcal/mol/A^2^), followed by simulations where the restraint was applied only to phosphate (P) atoms and Mg^2+^ ions with a force constant of 1 kcal/mol/A^2^), run for 5 ns. The ligands were stabilized by employing distance-to-bound-configuration (DBC) restraints^38^ through the Colvars library^48^, constraining their RMSD from the bound configuration. For DBC definition, all the ligands’ heavy atoms and the RNA heavy atoms within a 4 Å radius of the ligand were included. Temperature and pressure were controlled by the Bussi^66^ thermostat and Berendsen^67^ barostat, respectively. Van der Waals interactions utilized a 9 Å cut-off, while electrostatic interactions were treated by particle mesh ewald (PME)^68^ with a real-space cutoff of 7 Å. Induced dipoles were calculated with a Preconditioned Conjugate Gradient solver with a convergence tolerance of 1 × 10^−5^ Debye^69^.

The polyanionic nature of RNA attracts water molecules and counterions, influencing its stability. Additionally, Mg^2+^ ions can shed their hydration shells and establish strong interactions with specific sites in the RNA, promoting local structural rearrangements. To mitigate artifacts during the production phase, positional restraints were applied to the phosphate (P) atoms and Mg^2+^ ions with a force constant of 1 kcal/mol/A^2^). To prevent fraying, distance restraints were added to enforce the hydrogen bonds between the terminal base pairs. The partially restrained simulations were run for 40 ns for production; however, for all non-binders, simulations were run for 100 ns.

### DBC coordinate for binding pose determination and restraint definition

Accurate alchemical absolute free energy calculations require the use of restraints to ensure proper convergence. Typically, these restraints involve harmonic restraints between centers of mass of the ligand and the binding site (positional restraints)^70^ or more sophisticated methods like ’Boresch’ restraints^71^, which act on distances, angles, and dihedrals (positional and orientational restraints). In this study, we employed a more refined approach using DBC collective variables. DBC measures the RMSD of ligand coordinates relative to the moving frame of the receptor’s binding site^38^. This metric captures positional, orientational and conformational deviation of the ligand in a single collective variable. The restraint is flat-bottomed with a starting value that matches the distribution characteristic of the binding mode. This distribution can be efficiently monitored using the Colvars Dashboard^72^ within the VMD software^73^. Depending on the binding scenario, this distribution will be narrow for poses that are tightly bound, while it will be broader for systems with a diverse set of loosely bound configurations. The primary goal of these restraints is to restrict sampling during the decoupling process to those configurations that are relevant in the fully coupled state and thus facilitate convergence.

### lambda-ABF for ABFE simulations

As stated above, we have resorted here to alchemical free energy simulations in which the ligand is progressively “alchemically” decoupled from its environment, first in complex with the riboswitch host, and separately from the bulk solvent. The standard free energy of binding is obtained through a thermodynamic cycle. Such techniques require the sampling of the alchemical Hamiltonians. State-of-the-art methods typically use a predefined λ schedule with simulations at fixed λ values and a post-processing stage in which free energy differences are computed using estimators such as Thermodynamic Integration (TI)^74^ or Free Energy Perturbation^75^. However, these fixed-λ simulations can hinder the relaxation of orthogonal degrees of freedom that may benefit from a variable λ exploration which can be made possible through additional enhanced sampling techniques such as Hamiltonian Replica Exchange^76^ or Expanded Ensemble methods^77^. Here, we used the newly developed lambda-ABF approach^27^ which enables efficient relaxation of orthogonal degrees of freedom due to the variable nature of λ, as well as ergodic sampling thanks to the ABF bias. Its sampling efficiency has been shown to be superior compared to more traditional techniques. We utilized its implementation within Tinker-HP, in combination with the Colvars library^48^, which facilitates user-friendly convergence estimation without requiring post-processing, and allows integration with other CV-based methods.

For calculating the free energy surface of our systems at *T* = 300 K, we used four walkers with the lambda-ABF method and decoupled vdW and ELE interactions sequentially in separate legs. Each walker was run for 50 ns per leg. We ran three independent replicas for each setup. As for the free energy cost associated with the release of the DBC restraint, it is computed in the gas phase through TI by progressively releasing it to a compatible harmonic distance restraint which can then be computed analytically^27,38,53^.

During the alchemical decoupling, the phosphate atoms of the RNA backbone and the Mg^2+^ ions were restrained with a slight force constant of 1 kcal/mol/A^2^). It allows to, (i) prevent any destructuration, (ii) measure the ligand binding contribution independently from any Apo-Holo conformation changes by fixing the endpoint of the thermodynamic cycle.

Concerning the ion concentration used, it is well known that it can significantly impact the results of binding affinities^78^, especially for charged systems such as RNA-ligand complexes^79^. Reproducing all experimental conditions is crucial to get meaningful results in free energy simulations. However, methodological issues can arise when computing electrostatics using standard methods like PME in periodic boundary conditions. Although finite size effects diminish with larger simulation boxes, they may still be non-negligible in practice^80^. A notable issue occurs with PME in non-neutralized boxes which happen by default when scaling down electrostatics of ligands during alchemical simulations. In the reference experimental setup for mass spectrometry (MS) based binding assay that was used to measure the K_*D*_, the incorporation of a physiological ion concentration was not evoked.^42^ Given the positively charged nature of all the ligands reported here (2 to 3 positive charges) we cannot scale down the charge of anions to maintain a neutral charge of the simulation box in this setup. We have thus employed two distinct protocols for ABFE calculations:

- *Neutralized setup (Neut.):* K^+^ counter-ions were added to neutralize the system, which led to non neutral boxes in the full decoupling state (total charge of 2 to 3, depending on the ligand). This setup allows us to assess the impact of non neutral boxes and the absence of physiological ions on binding affinities.
- *Physiological Ion Concentration (Phys.):* 0.15 Molar of KCl were added to the system 2 to 3 Cl^−^ ions had their charge scaled down during ligand scaling (neutral boxes maintained). This setup aims at replicating physiological conditions more closely and evaluating the effect of physiological ionic strength on the computed binding affinities.

### OPES explore

OPES Explore^55^ is a CV-based enhanced sampling technique which is an evolution of metadynamics^81^. It aims at broadening the system’s sampling to a target probability distribution known as the Well-tempered distribution, defined as

$${p}^{{{{\rm{tg}}}}}({{{\bf{s}}}})\propto {\left[P({{{\bf{s}}}})\right]}^{\frac{1}{\gamma }}$$

where *P*(**s**) represents the unbiased marginal distribution of chosen CVs, and *γ* is the bias factor controlling the broadening.

To achieve this, Gaussian kernels are employed to reconstruct *p*^tg^(**s**), which in turn determines the bias potential *V*(**s**) through the following recursive strategy at step *n*:

$$V({{{\bf{s}}}})= 	 \, (\gamma -1){k}_{{{{\rm{B}}}}}T\log \left(\frac{{p}^{{{{\rm{tg}}}}}({{{\bf{s}}}})}{Z}+\epsilon \right)\\ {p}^{{{{\rm{tg}}}}}({{{\bf{s}}}})= 	 \, \frac{1}{n}{\sum}_{k=1}^{n}{G}_{k}({{{\bf{s}}}},{{{{\bf{s}}}}}_{k})$$

where *k*_B_ is the Boltzmann constant, *T* is the temperature set by the thermostat, *Z* is a normalization factor, and *G*_*k*_(**s**, **s**_*k*_) represents the Gaussian kernel deposited at step *k*.

The initial Gaussian kernel width, often denoted as SIGMA, typically corresponds to the standard deviation of **s** in the initial basin. The bias factor *γ* is typically set using *γ* = Δ*E*/(*k*_B_*T*), where Δ*E* is the barrier parameter. The regularization term *ϵ* ensures stability and is related to Δ*E* through $\epsilon ={{{{\rm{e}}}}}^{-\Delta E/({k}_{{{{\rm{B}}}}}T(1-1/\gamma ))}$.

The barrier parameter Δ*E* sets a limit on the maximum bias energy that OPES^54^ can introduce into the system. It should be large enough to facilitate transitions away from the initial basin while preventing the system from accessing irrelevant high-energy states that are difficult to reverse.

For further details, refer to the original literature introducing the OPES method^54,55^.

### Enhanced CV design using Deep-LDA

Deep-LDA^49^ is a way to define CV that correspond to a transition between two conformational states. It is rooted in LDA^82^, a classical technique in classification tasks. Here, it is tailored to differentiate between Apo and Holo states observed in unbiased system simulations.

Standard LDA optimizes Fisher’s ratio $\frac{{{{{\bf{w}}}}}^{T}{{{{\boldsymbol{S}}}}}_{{{{\rm{b}}}}}{{{\bf{w}}}}}{{{{{\bf{w}}}}}^{T}{{{{\boldsymbol{S}}}}}_{{{{\rm{w}}}}}{{{\bf{w}}}}}$, where **w** defines the discriminant direction *s*(*R*) = **w**^*T*^**d**(*R*). Here, ***S***_b_ and ***S***_w_ represent between- and within-class scatter matrices respectively, calculated from descriptor distributions in Apo and Holo states.

Deep-LDA extends LDA by employing a neural network (NN) to process *N*_d_ descriptors **d**, optimizing LDA within the last hidden layer **h**. The resulting Deep-LDA CV *s* = **w**^*T*^**h** offers a non-linear, expressive projection that has proven effective in various applications^83–85^. To enhance usability in simulations, we apply a cubic transformation, *s*_*w*_ = *s* + *s* ^3^^83,86^, ensuring smoother distributions.

### Statistics and reproducibility

All the free energy simulations performed in this study have been produced three times by varying the random seed that generates the initial velocities. The final results are the average of these three repeats and the standard deviation is calculated among these replicates. Each leg of the complex phase (riboswitch in complex with the ligands of interest) has been run for 50 ns and each leg of the solvent phase (where the ligand lies alone in bulk water) has been run for 20 ns.

### Reporting summary

Further information on research design is available in the Nature Portfolio Reporting Summary linked to this article.

## Supplementary information

Supplementary information file

Description of Additional Supplementary Files

Supplementary Data 1

Reporting Summary

## Data Availability

The input files used in this study are available on GitHub: https://github.com/krystel-elhage/targeting_rna. Numerical source data for graphs are provided as Supplementary Data 1; it contains the source data behind the graphs in Figures 4 and 6, and Supplementary Figs. S26, S27 and S28.

## Code availability

The code used during the study is available on GitHub: https://github.com/TinkerTools/tinker-hp, https://github.com/TinkerTools/poltype2, https://github.com/Colvars/colvars.
