# Supplementary material for: Targeting RNA with small molecules using state-of-the-art methods provides highly predictive affinities of riboswitch inhibitors
Source: Commun Biol. 2025 Oct 1;8:1405. doi: 10.1038/s42003-025-08809-y (PMC12488954; doi:10.1038/s42003-025-08809-y)
Supplement: Supplementary file 1 — Supplementary information file [file 42003_2025_8809_MOESM1_ESM.pdf]

Supplementary Information for "Targeting RNA with Small Molecules using State-of-the-Art Methods Provides Highly Predictive Affinities of Riboswitch Inhibitors"

Narjes Ansari,[*,†] Chengwen Liu,[†,‡] Florent Hédin,[†] Jérôme Hénin,[¶] Jay W. Ponder,[†,§] Pengyu Ren,[‡,†] Jean-Philip Piquemal,[‖,†] Louis Lagardère,[*,‖,†] and Krystel El Hage[*,†]

†*Qubit Pharmaceuticals, 75014 Paris, France*

‡*The University of Texas at Austin, Department of Biomedical Engineering, Austin, TX, USA*

¶*Université Paris Cité, CNRS, Laboratoire de Biochimie Théorique, 75005 Paris, France*

§*Washington University in Saint Louis, Department of Chemistry, Saint Louis, MO, USA*

‖*Sorbonne Université, Laboratoire de Chimie Théorique, UMR 7616 CNRS, 75005 Paris, France*

E-mail: narjesa@qubit-pharmaceuticals.com; louis.lagardere@sorbonne-universite.fr; krystel.elhage@qubit-pharmaceuticals.com

The Supplementary Information contains additional simulation details and amplified results/discussion regarding MD and ABFE simulations (Sections I-V), twenty eight supple-

mentary figures (supplementary Figs. S1-S28) and five supplementary tables (supplementary Tables S1-S5). Additional references are also provided.

I- Atoms involved in the definition of DBC for restraint and DBC correction

Atoms involved in the definition of DBC for restraint in L-ABF of the complex phase are displayed in Fig. S1. Atoms chosen from the ligand are highlighted in yellow, corresponding to the main scaffold of the ligands, while atoms from the RNA are shown as red spheres. The indices of bases and corresponding atoms are colored similarly. Additionally, Table S1 reports the corrections corresponding to the DBC and Harmonic restraints.

Figure S1: **Atoms Involved in the Definition of DBC for Restraint**.

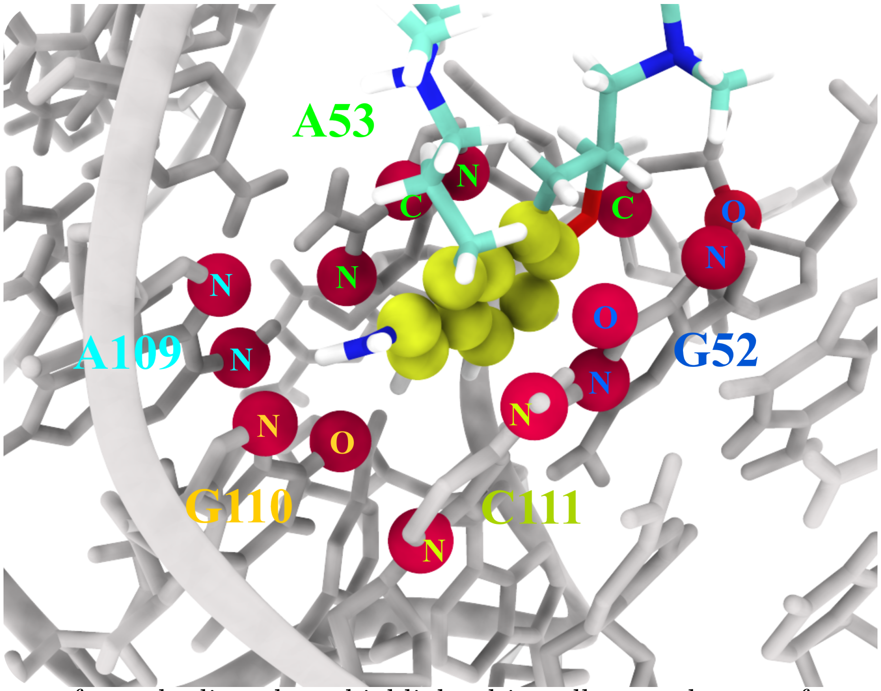

Atoms chosen from the ligand are highlighted in yellow, and atoms from the RNA are shown as red spheres. The bases and corresponding atoms are colored in the same manner.

Table S1: Amount of restraint from Distance-to-Bound-Conformation (DBC) and Harmonic (H) restraints (in kcal/mol) for the six representative ligands obtained from lambda-ABF. The values are calculated over three replicas, and the error corresponds to the standard deviation over these replicas.

**Ligand**	**DBC**	**Harmonic**
**1**	2.91 $\pm$ 0.02	3.4
**2**	2.90 $\pm$ 0.02	3.4
**3**	3.71 $\pm$ 0.03	4.28
**4 (SS0)**	2.89 $\pm$ 0.02	3.4
**5**	2.90 $\pm$ 0.02	3.4
**6**	2.30 $\pm$ 0.02	3.4

II- ELE and vdW decomposition of PMF from ABFE simulations

Here we present the electrostatic (ELE) and van der Waals (vdW) decomposition of the Potential of Mean Force (PMF) of the six representative ligands (ligand 1 to 6) along with convergence plots for both the neutralized system and the system under physiological ion concentration (supplementary Figs.S2-S25). For each ligand, three replicas are reported.

Table S2: ELE and vdW decomposition of the Potential of Mean Force (PMF) for the complex, solvent, and gas phases of the neutralized setup of Ligands 1 to 6. For each ligand, three replicas are reported. The energies are reported in kcal/mol.

		**Complexation**		**Solvation**		**Gas**	
**Ligand**	**replica**	**ELE**	**vdW**	**ELE**	**vdW**	**ELE**	**vdW**
**1**	**1**	-77.39	31.21	-60.78	30.32	-82.51	24.00
	**2**	-78.45	30.56	-60.70	30.22	-82.49	24.88
	**3**	-78.81	30.94	-60.79	30.13	-82.60	24.83
**2**	**1**	-74.87	22.63	-58.48	30.32	-84.07	24.88
	**2**	-75.11	23.82	-58.57	30.27	-83.98	24.88
	**3**	-74.66	23.54	-58.53	30.26	-83.99	24.83
**3**	**1**	-105.60	37.54	-86.34	40.65	-177.89	32.99
	**2**	-105.62	36.47	-86.36	40.51	-177.85	32.84
	**3**	-105.83	34.83	-86.29	40.61	-177.97	33.11
**4**	**1**	-68.05	35.73	-51.83	43.33	-223.17	35.39
	**2**	-68.55	36.78	-51.79	43.18	-223.11	35.38
	**3**	-69.02	35.94	-51.90	43.29	-223.23	35.47
**5**	**1**	-98.65	43.89	-82.95	51.47	-175.31	45.03
	**2**	-98.89	43.02	-82.94	51.88	-175.39	44.83
	**3**	-98.06	43.47	-82.88	51.91	-175.04	44.83
**6**	**1**	-67.60	41.77	-46.65	48.80	-227.85	41.89
	**2**	-67.19	43.14	-46.62	48.63	-227.88	46.20
	**3**	-66.57	43.79	-46.66	48.80	-227.87	41.88

Table S3: ELE and vdW decomposition of PMF for complex, solvent, and gas phases. The energies are reported in kcal/mol. The system is in physiological ion concentration of Ligands 1 to 6. For each ligand, three replicas are reported.

		**Complexation**		**Solvation**		**Gas**	
**Ligand**	**replica**	**ELE**	**vdW**	**ELE**	**vdW**	**ELE**	**vdW**
**1**	**1**	-255.87	34.03	-238.47	35.76	-82.51	24.00
	**2**	-255.10	33.76	-238.39	35.76	-82.49	24.88
	**3**	-255.92	35.88	-238.42	35.83	-82.60	24.83
**2**	**1**	-251.48	26.56	-236.05	36.06	-84.07	24.88
	**2**	-251.06	27.82	-236.01	36.17	-83.98	24.88
	**3**	-251.46	26.86	-236.00	36.10	-83.99	24.83
**3**	**1**	-374.62	43.52	-352.99	48.65	-177.89	32.99
	**2**	-373.76	44.07	-352.80	48.64	-177.85	32.84
	**3**	-373.52	43.96	-352.93	48.79	-177.97	33.11
**4**	**1**	-336.50	42.30	-318.47	51.75	-223.17	35.39
	**2**	-336.18	43.55	-318.40	51.72	-223.11	35.38
	**3**	-336.34	45.01	-318.40	51.87	-223.23	35.47
**5**	**1**	-364.86	52.31	-349.38	60.23	-175.31	45.03
	**2**	-363.94	51.64	-349.65	60.46	-175.39	44.83
	**3**	-364.55	52.06	-349.53	60.51	-175.04	44.83
**6**	**1**	-333.65	50.55	-313.24	57.54	-227.85	41.89
	**2**	-333.15	50.18	-313.12	57.61	-227.88	46.20
	**3**	-333.53	50.56	-313.12	57.66	-227.87	41.88

Ligand 1

Figure S2: **Convergence plots of ELE and vdW legs for complex phase in the neutralized (Neut.) setup for Ligand 1.**

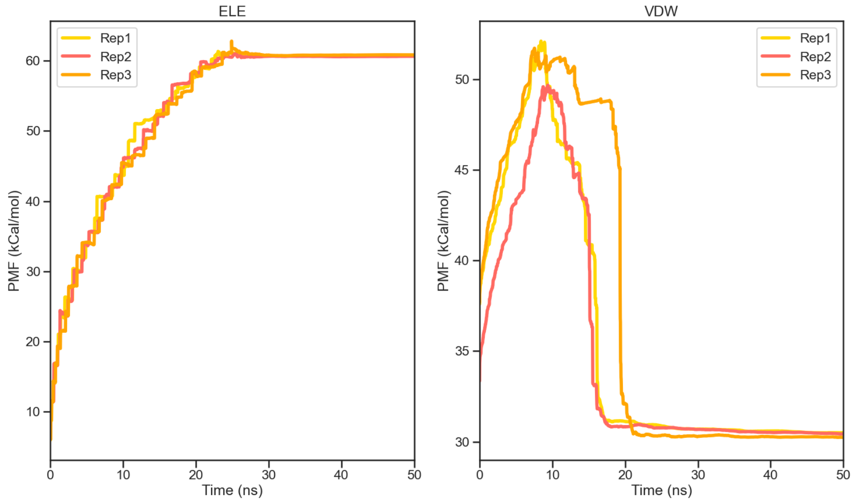

Figure S3: **Convergence plots of ELE and vdW legs for solvent phase in the neutralized (Neut.) setup for Ligand 1.**

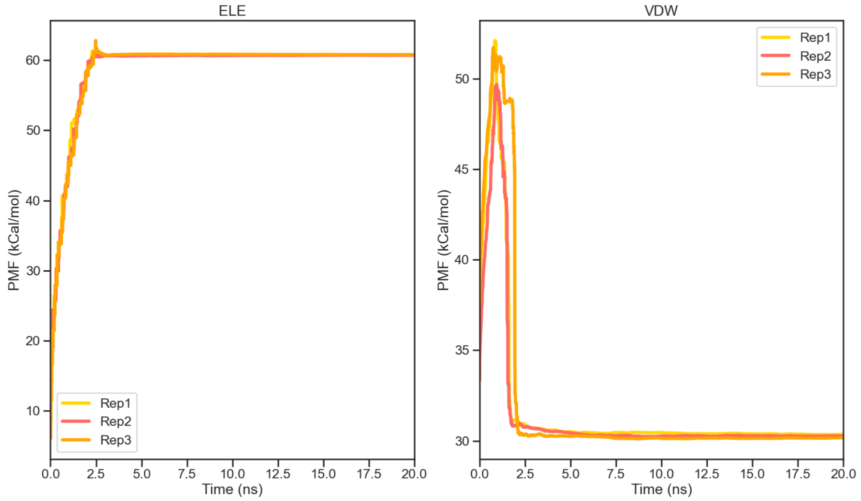

Figure S4: **Convergence plots of ELE and vdW legs for complex phase in the physiological ion concentration (Phys.) setup for Ligand 1**.

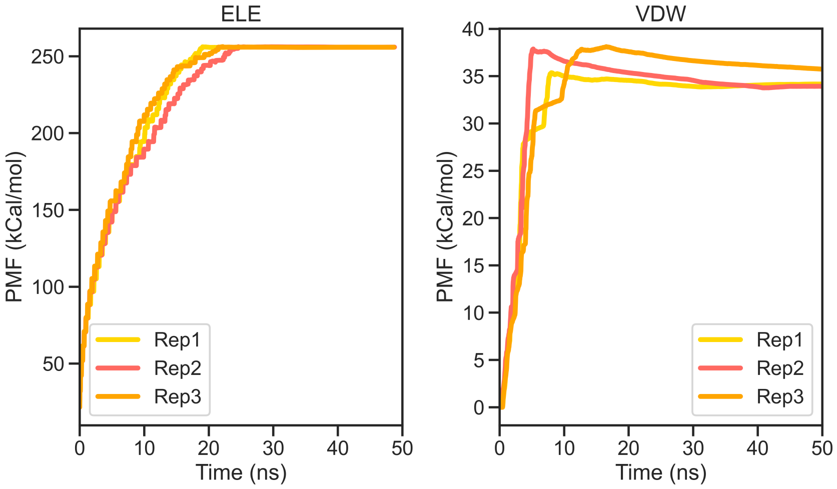

Figure S5: **Convergence plots of ELE and vdW legs for solvent phase in the physiological ion concentration (Phys.) setup for Ligand 1.**

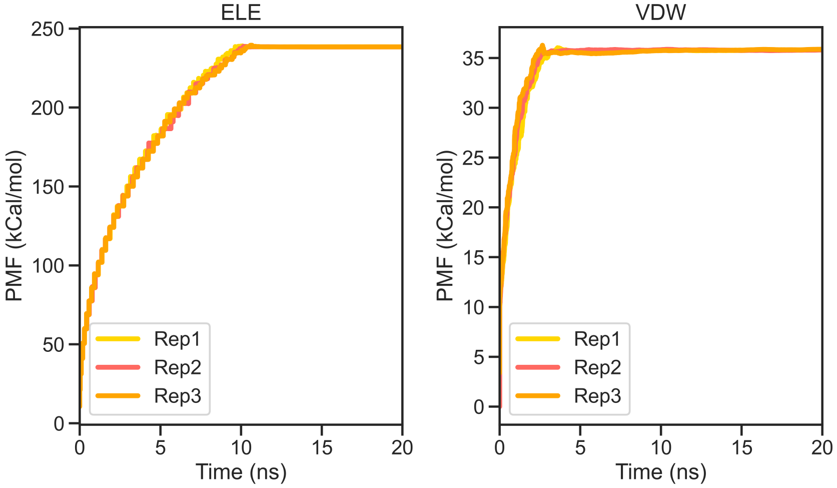

Ligand 2

Figure S6: **Convergence plots of ELE and vdW legs for complex phase in the neutralized (Neut.) setup for Ligand 2**.

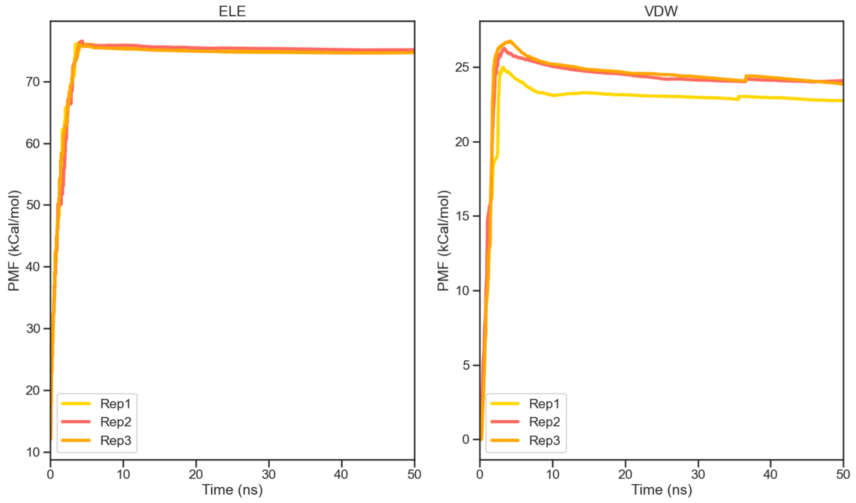

Figure S7: **Convergence plots of ELE and vdW legs for solvent phase in the neutralized (Neut.) setup for Ligand 2**.

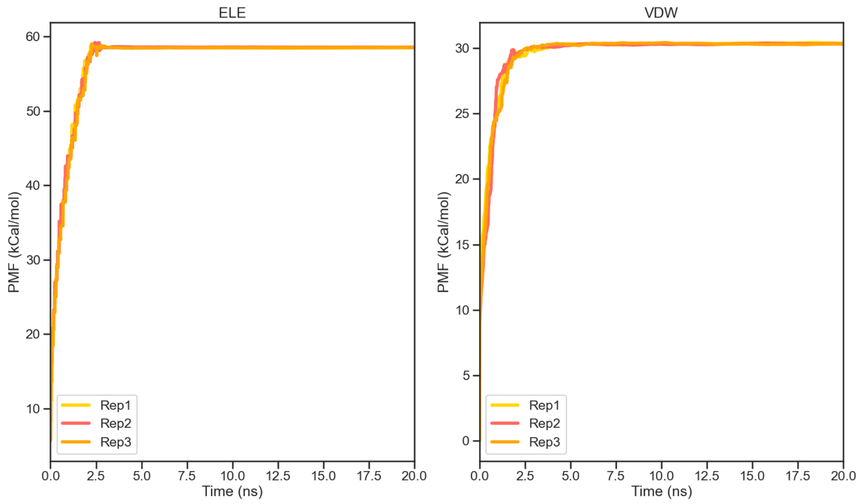

Figure S8: **Convergence plots of ELE and vdW legs for complex phase in the physiological ion concentration (Phys.) setup for Ligand 2.**

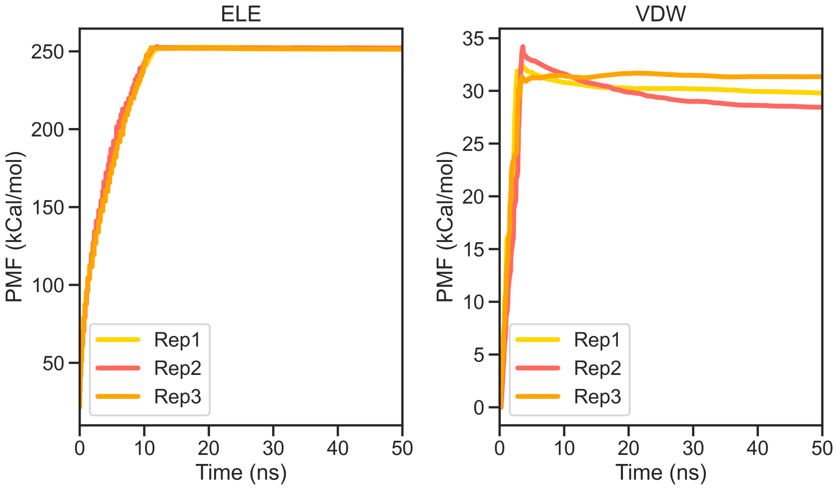

Figure S9: **Convergence plots of ELE and vdW legs for solvent phase in the physiological ion concentration (Phys.) setup for Ligand 2.**

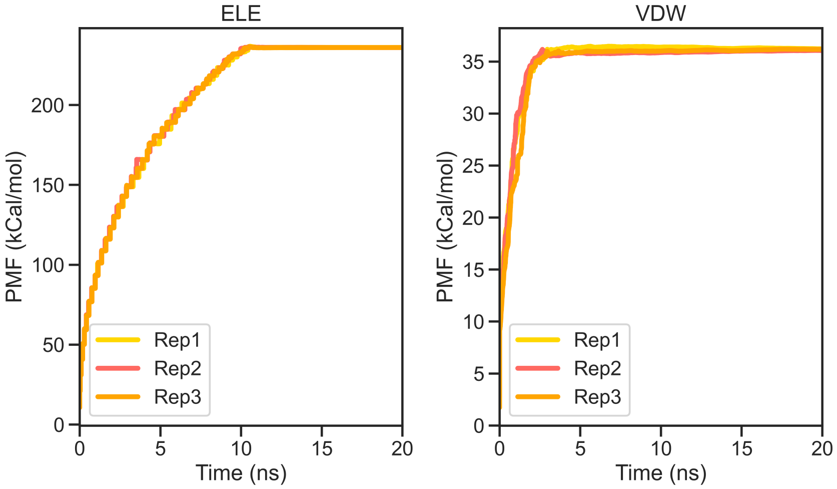

Ligand 3

Figure S10: **Convergence plots of ELE and vdW legs for complex phase in the neutralized (Neut.) setup for Ligand 3**.

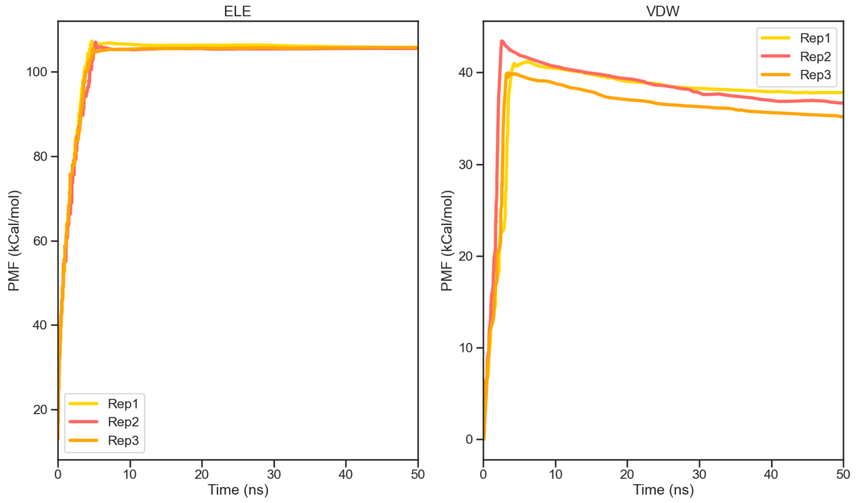

Figure S11: **Convergence plots of ELE and vdW legs for solvent phase in the neutralized (Neut.) setup for Ligand 3**.

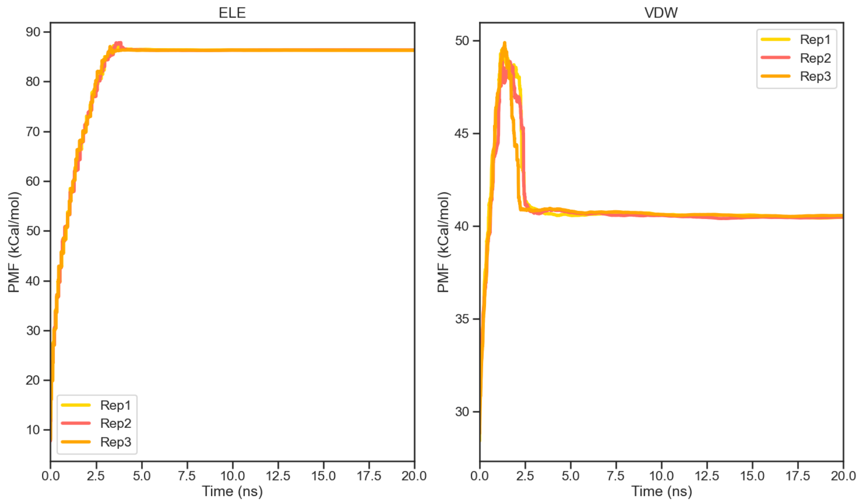

Figure S12: **Convergence plots of ELE and vdW legs for complex phase in the physiological ion concentration (Phys.) setup for Ligand 3**.

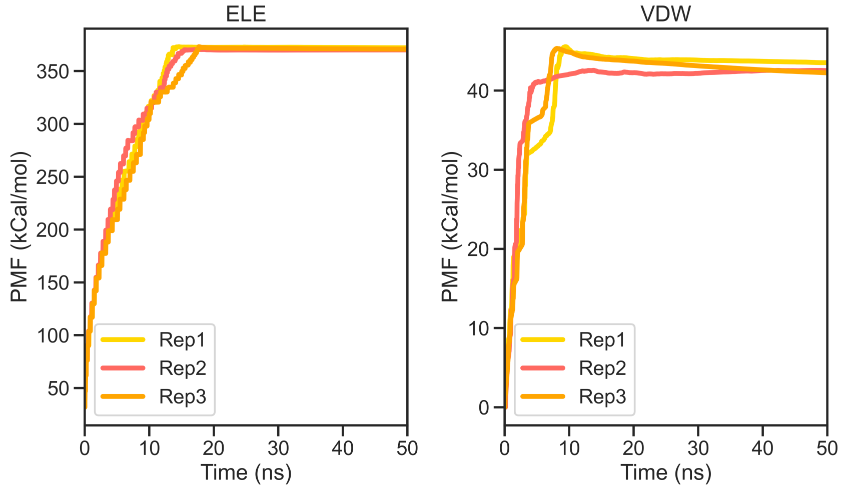

Figure S13: **Convergence plots of ELE and vdW legs for solvent phase in the physiological ion concentration (Phys.) setup for Ligand 3**.

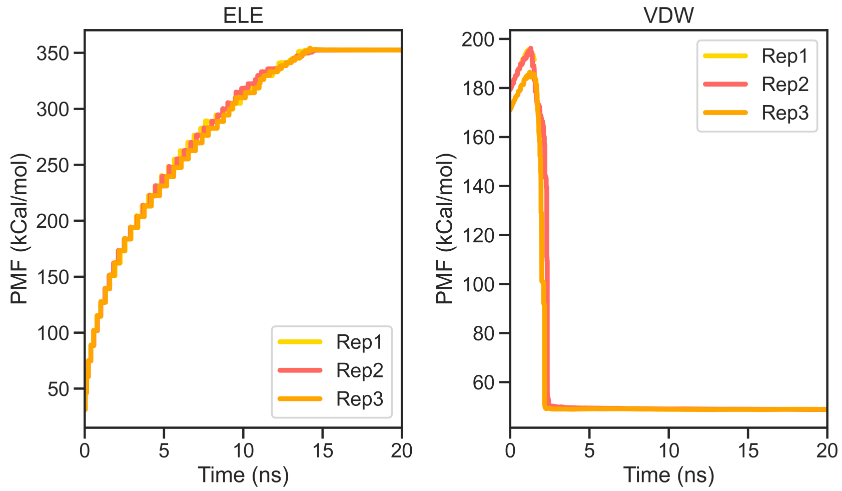

Ligand 4

Figure S14: **Convergence plots of ELE and vdW legs for complex phase in the neutralized (Neut.) setup for Ligand 4.**

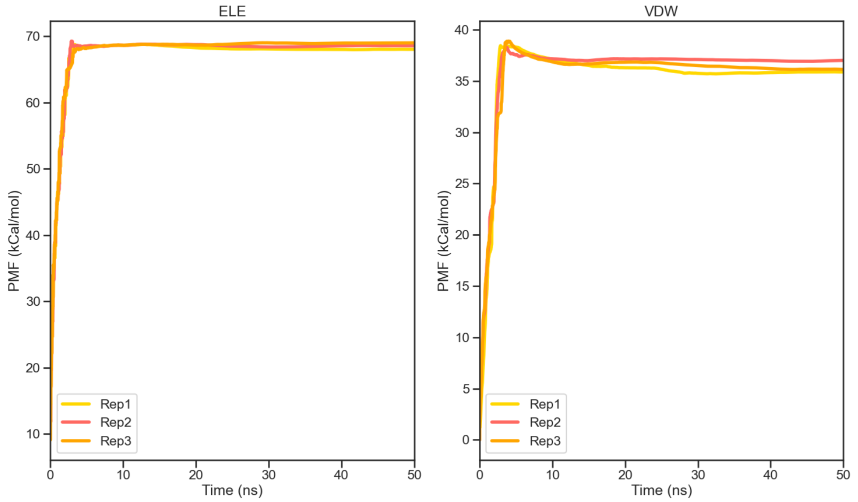

Figure S15: **Convergence plots of ELE and vdW legs for solvent phase in the neutralized (Neut.) setup for Ligand 4.**

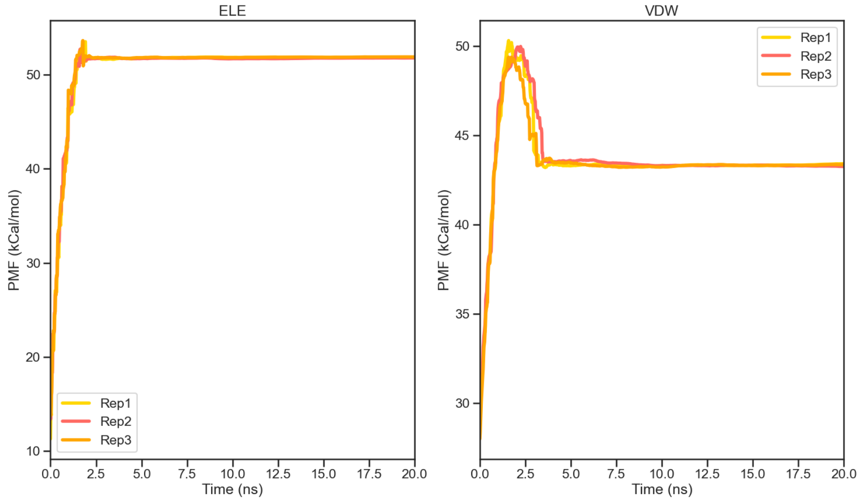

Figure S16: **Convergence plots of ELE and vdW legs for complex phase in the physiological ion concentration (Phys.) setup for Ligand 4.**

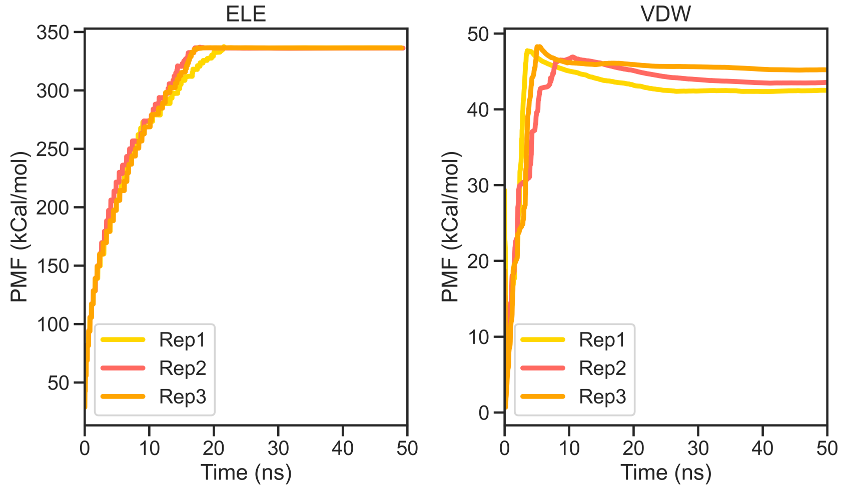

Figure S17: **Convergence plots of ELE and vdW legs for solvent phase in the physiological ion concentration (Phys.) setup for Ligand 4.**

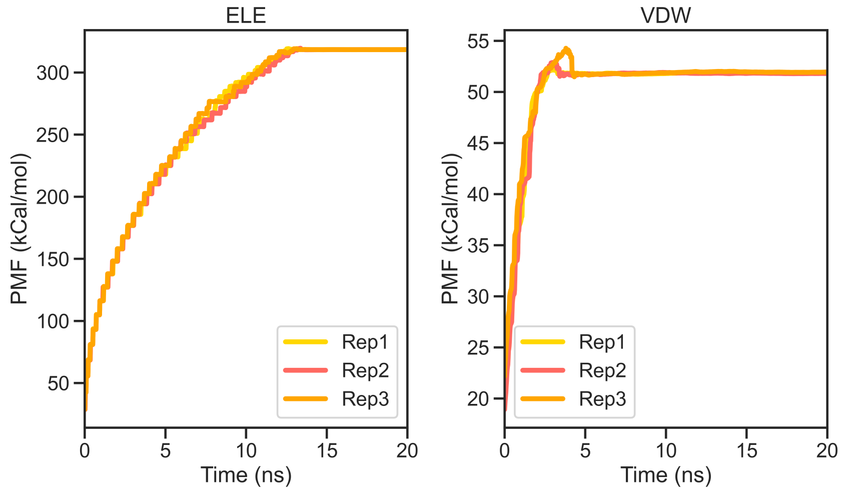

Ligand 5

Figure S18: **Convergence plots of ELE and vdW legs for complex phase in the neutralized (Neut.) setup for Ligand 5**.

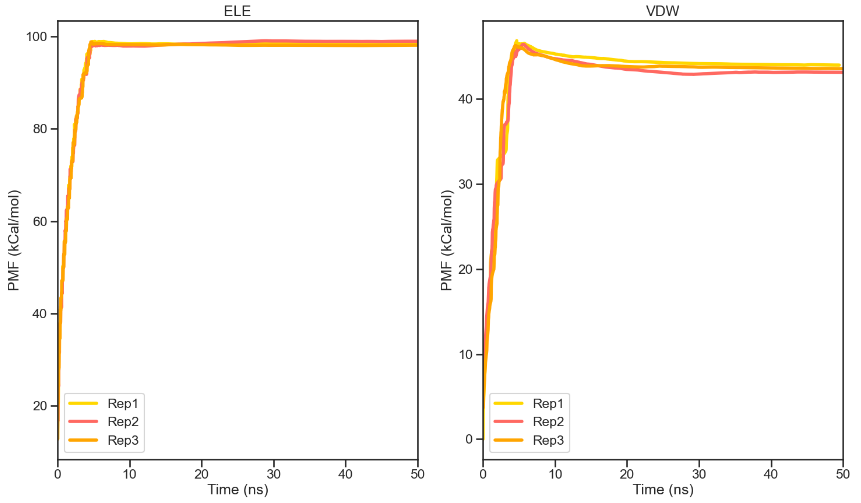

Figure S19: **Convergence plots of ELE and vdW legs for solvent phase in the neutralized (Neut.) setup for Ligand 5**.

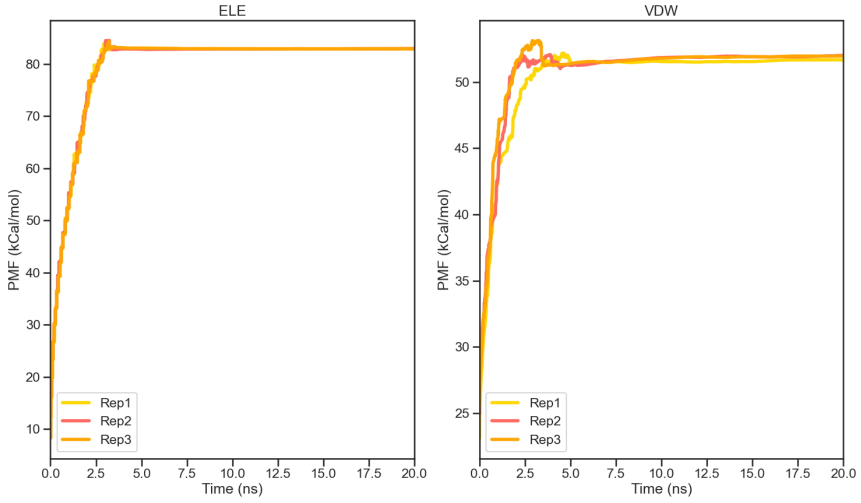

Figure S20: **Convergence plots of ELE and vdW legs for complex phase in the physiological ion concentration (Phys.) setup for Ligand 5.**

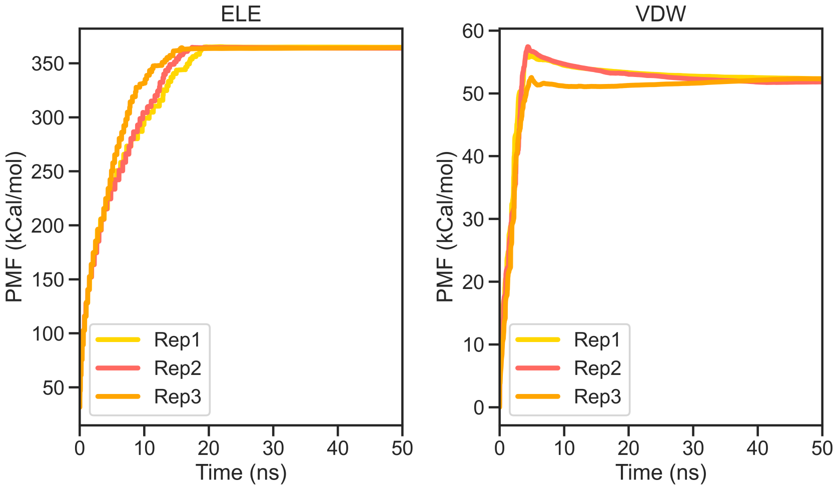

Figure S21: **Convergence plots of ELE and vdW legs for solvent phase in the physiological ion concentration (Phys.) setup for Ligand 5.**

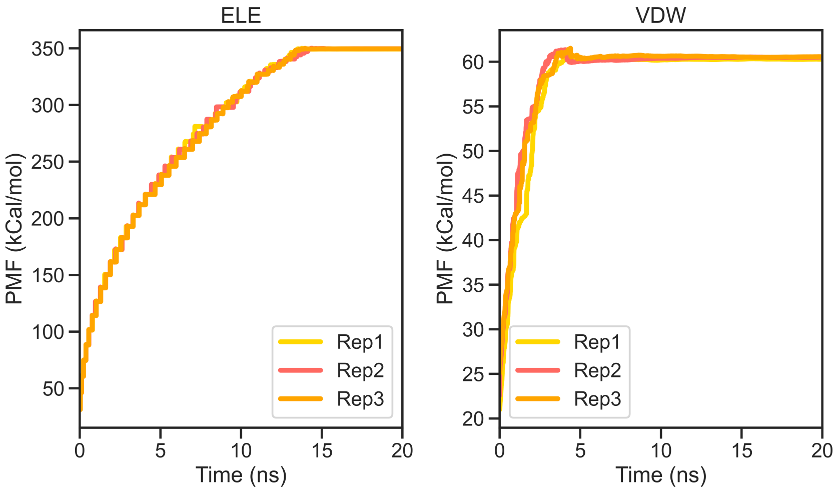

Ligand 6

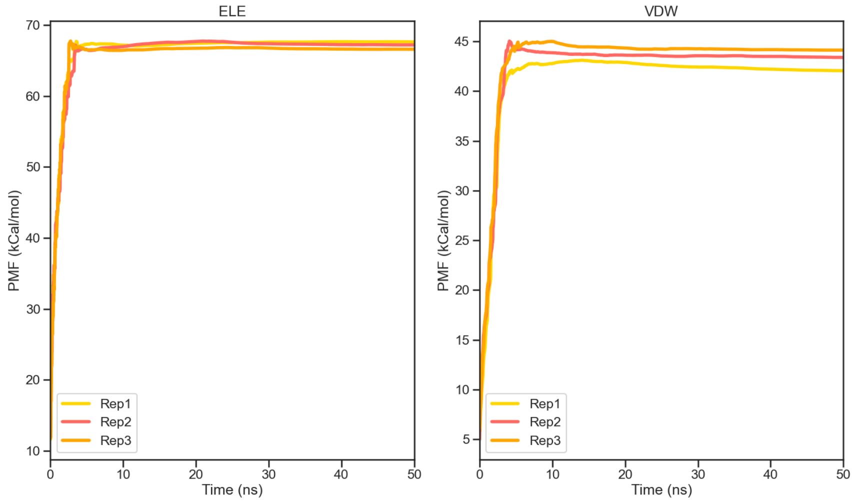

Figure S22: **Convergence plots of ELE and vdW legs for complex phase in the neutralized (Neut.) setup for Ligand 6**.

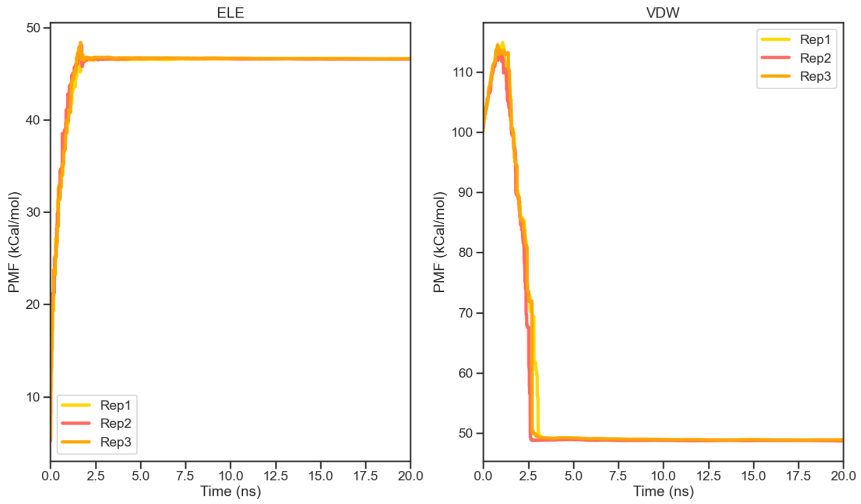

Figure S23: **Convergence plots of ELE and vdW legs for solvent phase in the neutralized (Neut.) setup for Ligand 6**.

Figure S24: **Convergence plots of ELE and vdW legs for complex phase in the physiological ion concentration (Phys.) setup for Ligand 6**.

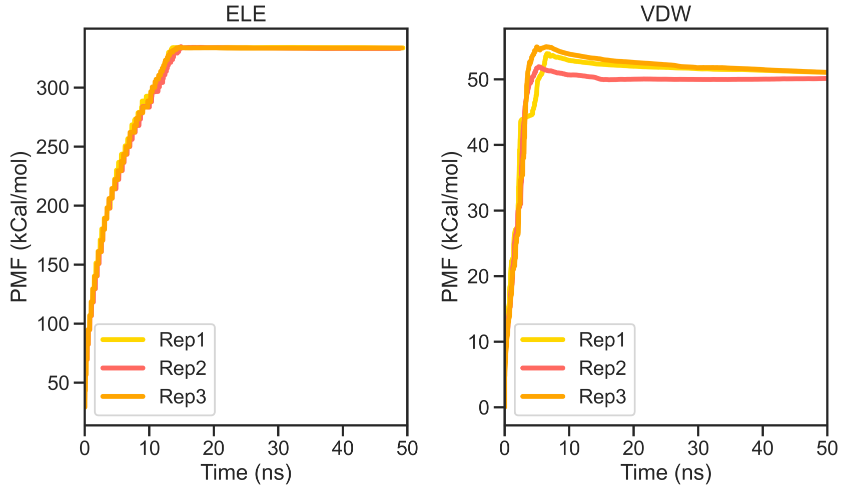

Figure S25: **Convergence plots of ELE and vdW legs for solvent phase in the physiological ion concentration (Phys.) setup for Ligand 6**.

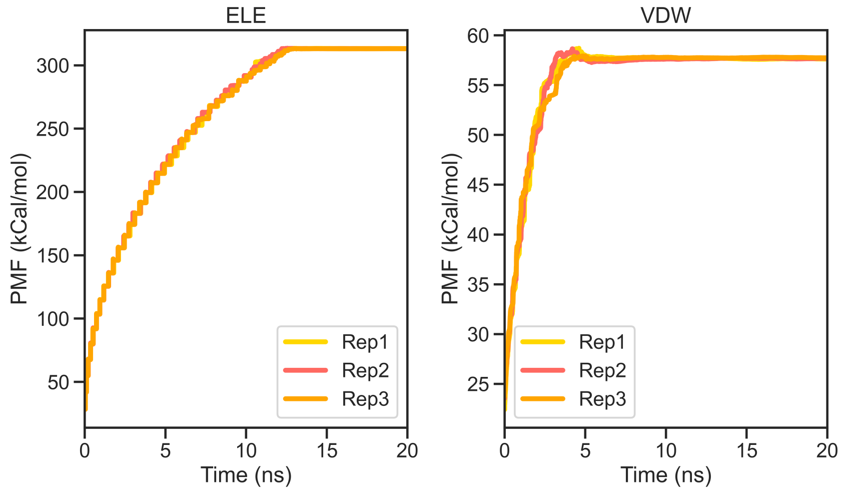

III- Simulation setup with a physiological ion concentration

Apart from the neutralized system setup (Neut.) for ABFE calculations, a second setup that includes a physiological ion concentration (Phys.) was employed for six representative ligands (Ligand 1 to 6). Table S4 reports the raw computed values ($\mathbf{\Delta G}_{\text{Comp-raw}}$) for each setup as well as the values after the apo/holo shift correction ($\mathbf{\Delta G}_{\text{Comp-Neut}}$ for the neutralized condition and ($\mathbf{\Delta G}_{\text{Comp-Phys}}$) in the presence of a physiological ion concentration. This shift yield a value of 10.35 $\pm$ 0.91 kcal/mol for the neutral setup and 11.33 $\pm$ 1.19 kcal/mol in the presence of physiological ion concentration. Despite this slight difference, the results are consistent within the error margins.

The correlation plots are illustrated in Fig. S26. Upon including the systematic shift between the calculated and experimental binding free energies to accommodate for the Apo-Holo protein reorganization free energy, we observe a correlation between computed and experimental results across the representative 6 ligands of 0.7 (Pearson r), with a root mean square error (RMSE) of 0.81 kcal/mol and a mean absolute error (MAE) of 0.70 kcal/mol for the neutralized setup (Fig. S26a). For the setup with a physiological ion concentration, a correlation of 0.7 with an RMSE of 1.05 kcal/mol and an MAE of 0.84 kcal/mol was observed (Fig. S26b). Both setups show similar results; a solid reproduction of the binding mode and a good ranking, with ligand 6 and ligand 4 identified as the most potent and ligand 1 as a non-binder.

Table S4: **Experimental and computed ΔG values of the six representative ligands.** Computed ΔGs are reported for two different conditions (in kcal/mol): neutralized complex ($\Delta G_{Comp-Neut}$) and including physiological ion concentration ($\Delta G_{Comp-Phys}$). For Ligands 4, 5, and 6, the experimental ΔG_{Exp} is measured on a 40-mer RNA, while for the rest, the 29-mer is used. All computed ΔG were measured using a 29-mer construct. ΔG_{Comp} and errors represent the mean and standard error of the mean from three replicas for each ligand. The raw data for computed ΔG ($\Delta G_{Comp-raw}$) are reported in parentheses. ΔG values are reported in kcal/mol. K_D values are reported in µM.

Ligand	K_D	ΔG_{Exp}	$\Delta G_{Comp-Neut}$($\Delta G_{Comp-raw}$)	$\Delta G_{Comp-Phys}$($\Delta G_{Comp-raw}$)
1	>200	>-5.05	-0.07 (-10.43) ± 0.92	-0.76 (-12.08) ± 0.88
2	40	-6.01	-6.61 (-16.96) ± 0.68	-6.67 (-18.00) ± 0.83
3	8	-6.69	-6.53 (-16.88) ± 1.54	-6.58 (-17.90) ± 0.75
4	0.86	-8.27	-7.13 (-17.48) ± 0.65	-8.42 (-19.75) ± 1.35
5	3.5	-7.44	-7.21 (-17.56) ± 0.81	-5.66 (-16.98) ± 0.19
6	0.72	-8.39	-9.60 (-19.95) ± 1.54	-9.75 (-21.08) ± 0.05

Figure S26: **Experimental vs Computed ΔG values of six representative ligands.**

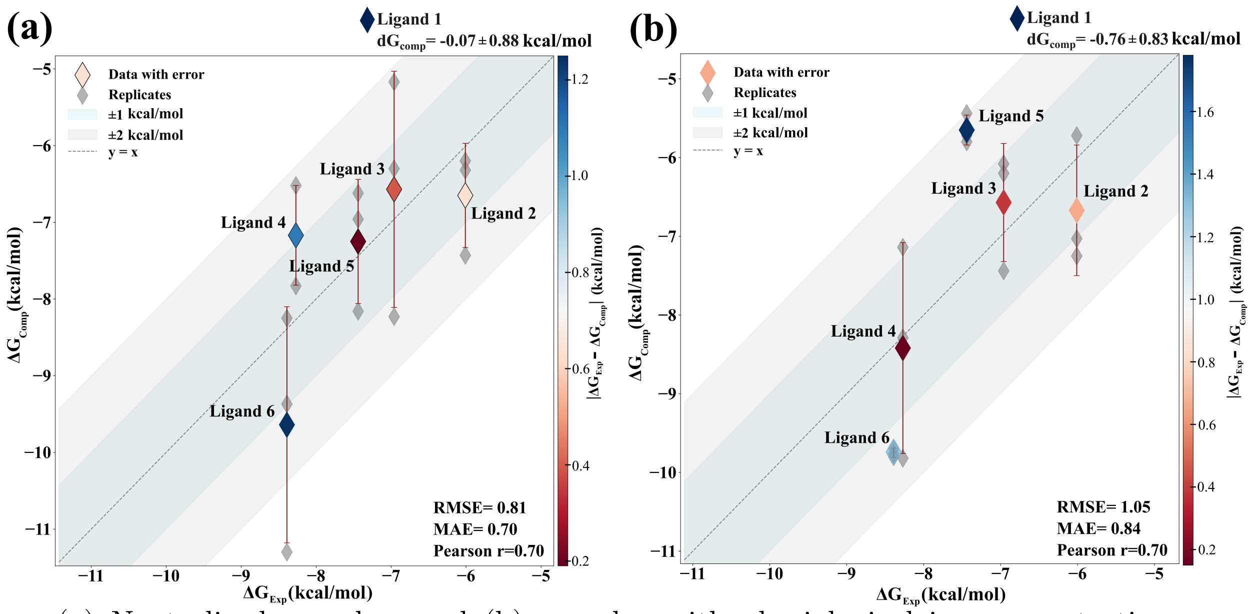

(a) Neutralized complex, and (b) complex with physiological ion concentration. Computed ΔG and errors are the mean and standard error of the mean from three replicas for each ligand. The dark shaded region spans ± 1 kcal/mol; the lighter region spans ± 2 kcal/mol. The color bar represents the absolute value difference between experimental and computed values. Pearson r, RMSE, and MAE are reported in the right bottom corner of each plot. The ΔG value of non-binder ligand 1 is reported on the top right of each panel.

IV- Performance of lambda-ABF for ABFE calculations on different GPU types

In ABFE simulations, the ligand is progressively "alchemically" decoupled from its environment, first in complex with the target (here RNA), and separately from the bulk solvent. The benchmark is done using Tinker-HP and the AMOEBA FF on three different GPU architectures: NVIDIA H100, A100 and V100. Table S5 reports the simulation time (ns/day) for a system size of 51'400 atoms, corresponding to an average systems from our RNA-ligand complexes, across the used GPUs, considering 1 walker for both ELE and vdW legs of the complex phase simulation (most computationally expensive phase of an ABFE calculation). On one hand, with the H100 GPU, we achieve approximately 24 and 19 ns/day for the vdW and ELE legs, respectively. On the other hand, the performance is reduced on A100 and V100 GPUs due to differences in computational power and memory bandwidth. On A100 GPUs, we achieve 15 and 12 ns/day for the vdW and ELE legs, respectively; and 10 and 8 ns/day on V100 GPUs for vdW and ELE legs, respectively. The performance on H100 GPUs is 1.5 times faster than A100 and 2.4 times faster than V100 GPUs. All in all, the total amount of simulation time for the present study is (4+4)*50*3*19=22800 ns (4 walker for the Ele leg, 4 walkers for the vdW leg, 50 nanoseconds per walker, 3 repeats per system, 19 systems.

Table S5: Performance of complex phase simulations on different GPU architectures. The system size used corresponds to an average RNA-ligand complex taken from this study. The benchmark is done considering 1 walker for both ELE and vdW legs of the complex phase simulation.

System size (atoms)	Leg	V100 (ns/day)	A100 (ns/day)	H100 (ns/day)
51400	ELE	8	12	19
	vdW	10	14.8	24

V- Apo/Holo Conformational Change

As discussed in the main text, there is a significant free energy barrier between the Apo and Holo states. Since this conformational change involves a collective motion of the RNA, estimating the free energy barrier using CV-based enhanced sampling requires a CV capable of capturing this transformation. Simple CVs, such as distances or dihedrals, are insufficient for this system. Instead, more advanced machine learning-based CVs can be employed. To estimate the free energy difference, we employed OPES-EXPLORE in conjunction with Deep-LDA machine learning collective variables (CVs). The Deep-LDA strategy employed in this work is analogous to the approach presented in Bonati *et al.* [1]. The code for the training of the Deep-LDA is available through the open-source mlcolvar library [2]. To train the neural network (NN), we selected 47 descriptors that effectively distinguish between the Apo and Holo states. These descriptors are based on 20 different distances and 27 angles between phosphorus (P) and/or magnesium (Mg) atoms. The NN architecture consists of a sequence of layers with $28, 20, 30, 5$ nodes, using the rectified linear unit (ReLU) as the activation function. Training was performed using unbiased simulations of approximately 40 ns for both Apo and Holo states. The number of configurations per state used for training was around 5000. We employed Deep-LDA parameters: $\lambda = 0.05$, $\alpha = 2/\lambda$, and $\gamma = 10^{-5}$. The model was optimized using the ADAM optimizer with a learning rate of 2.5×10^{-5}. After training the NN, we used the 1D Deep-LDA CV for OPES-explore simulation. We set the barrier height around 15 kcal/mol and used an OPES-explore PACE of 500. Figure S27 shows the RMSD of the biased trajectory, illustrating the transition from the Apo to the Holo states. The RMSD values corresponding to Apo and Holo from plain MD simulations are represented by dashed lines. The corresponding Holo state after OPES simulation is illustrated in Figure S27b. Additionally, Figure S28 displays the approximate 1D free energy profile along the Deep-CV.

Figure S27: **RMSD evolution as a function of time of RNA, including the three Mg^{2+} ions, in simulations where the OPES explore barrier guess was set to 15 kcal/mol.**

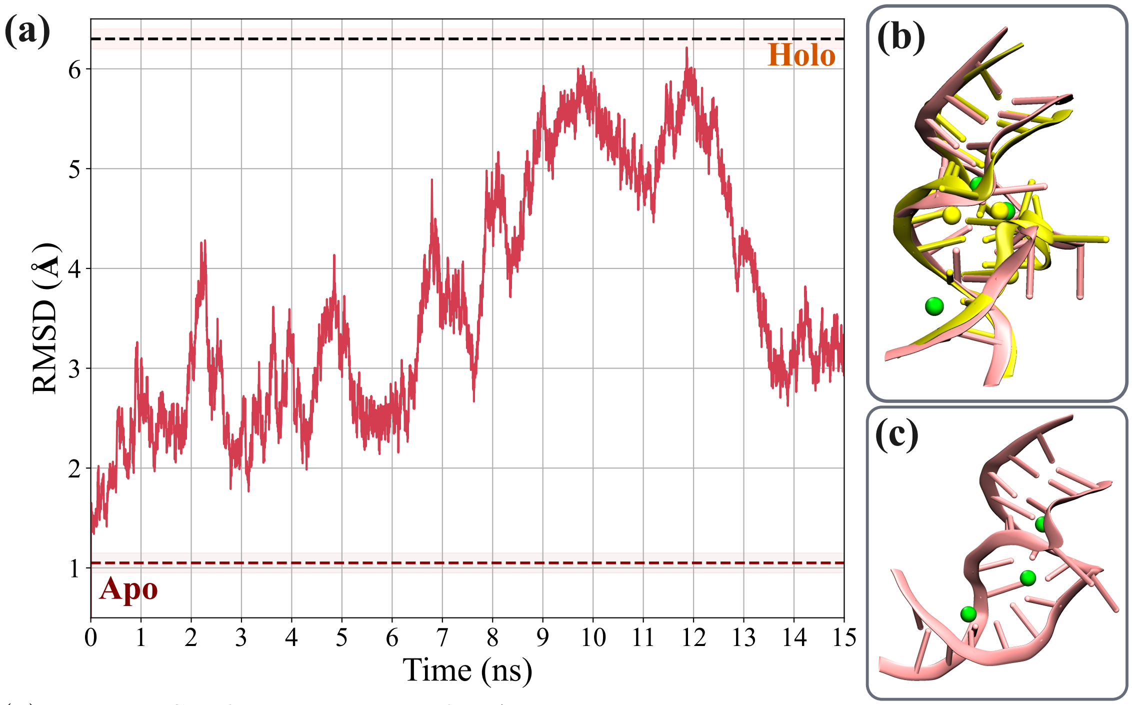

(a) The RMSD from plain MD for Apo and Holo states is indicated with dashed lines, with plain MD simulations running for 40 ns. (b) overlay of the x-ray Holo structure (yellow) to the Holo structure obtained after enhanced sampling simulations (pink). (c) Structure of the Apo state.

Figure S28: **The free energy landscape of Apo-Holo conformational changes.**

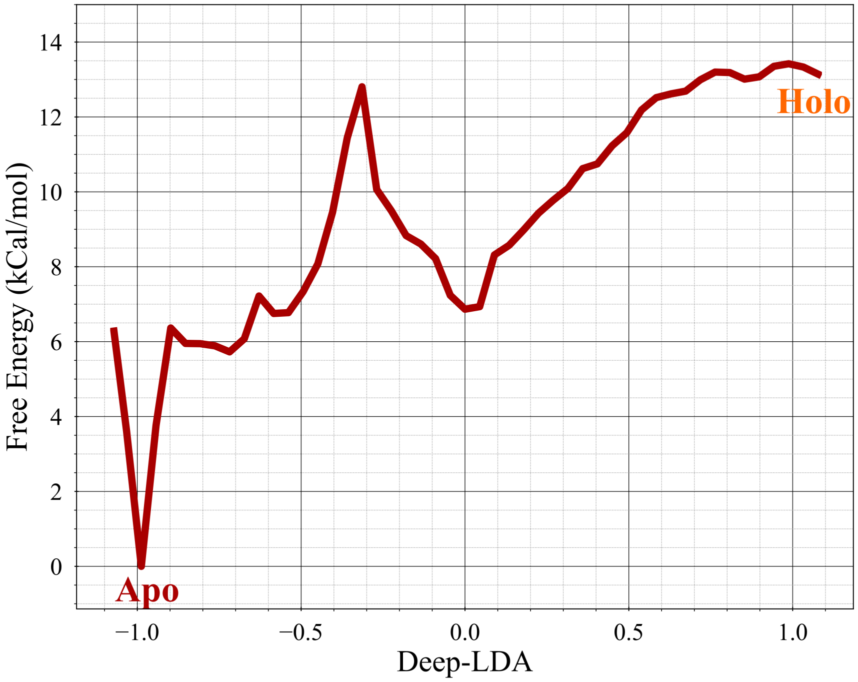

References

1. Bonati, L., Rizzi, V. & Parrinello, M. Data-driven collective variables for enhanced sampling. *J. Phys. Chem. Lett.* **11,** 2998–3004 (2020).

2. Bonati, L., Trizio, E., Rizzi, A. & Parrinello, M. A unified framework for machine learning collective variables for enhanced sampling simulations: mlcolvar. *J. Chem. Phys.* **159,** 014801 (2023).
