# Supplementary material for: Targeting RNA with small molecules using state-of-the-art methods provides highly predictive affinities of riboswitch inhibitors
Source: Commun Biol. 2025 Oct 1;8:1405. doi: 10.1038/s42003-025-08809-y (PMC12488954; doi:10.1038/s42003-025-08809-y)
Supplement: Supplementary file 2 — Description of Additional Supplementary Files [file 42003_2025_8809_MOESM2_ESM.pdf]

Description of Additional Supplementary Files

File name: Supplementary Data 1

Description: Numerical source data for graphs are provided as Supplementary Data 1 in Excel format. It contains the source data behind the graphs in the paper (Figures 4 and 6, and Supplementary Figures S26, S27 and S28).
